# Dual-Active Nanoimmunomodulators for the Synergistic Enhancement of the Antitumor Efficacy of Photodynamic Immunotherapy

**DOI:** 10.34133/bmr.0214

**Published:** 2025-06-09

**Authors:** Ping Dong, Shaowen Zhang, Ying Zhang, Haifeng Hu, Qing Zhou, Yanzhuo Liu, Zhangfan Mao

**Affiliations:** ^1^ Department of Thoracic Surgery, Renmin Hospital of Wuhan University, Wuhan, 430060 Hubei, People’s Republic of China.; ^2^ Department of Vascular Surgery, Renmin Hospital of Wuhan University, Wuhan, 430060 Hubei, People’s Republic of China.; ^3^ Department of Neurology, Tongji Hospital Affiliated to Tongji Medical College, Huazhong University of Science and Technology, Wuhan 430030, People’s Republic of China.; ^4^ Department of Pharmacology, Renmin Hospital of Wuhan University, Wuhan 430060, People’s Republic of China.

## Abstract

Photodynamic immunotherapy, which combines photodynamic therapy (PDT) with immunotherapy, has become an important and effective treatment for cancer. However, most photodynamic immunotherapy systems for cancer do not allow for the precise release of immunomodulators, leading to systemic side effects and poor patient prognosis. This study reports a dual-activatable nanoimmunomodulator (CPPM), whose photodynamic effect and agonist release are both activated in response to specific stimuli, which can be used for precise photodynamic immunotherapy of cancer. CPPM has a half-life of 119 min in circulation and accumulates in tumor tissue 4 h after injection (23.8%). In addition, CPPM is able to achieve tumor localization of nanomedicines through PD-L1-targeting peptides, blocking the specific binding of PD-L1 to PD-1, exposing tumor surface antigens, and reinvigorating the activity of T cells in combination with macitentan to promote T-cell proliferation. Meanwhile, under laser irradiation, CPPM was able to increase intracellular oxidative stress, inhibit cell proliferation through PDT, and trigger immunogenic cell death, further enhancing tumor immunogenicity through synergistic treatment. Ultimately, CPPM enhanced the immunotherapeutic efficiency against tumors by improving the tumor immunosuppressive microenvironment, synergistically inhibiting the growth of primary and distant tumors while activating systemic antitumor immunity to eliminate lung metastases without obvious side effects. This study presents an uncomplicated and multifunctional strategy for the precise modulation of tumor photodynamic immunotherapy with a dual-activatable smart nanoimmunomodulator that can improve the efficacy of PDT, enhance systemic antitumor immunity, and potentially extend it to a wide range of cancers.

## Introduction

Cancer is one of the leading causes of death globally, with nearly 20 million new cancer cases and about 9.7 million deaths in 2022 worldwide [1]. Currently, there are also more and more treatments for cancer, but traditional antitumor drugs have low drug utilization, poor biocompatibility, and high side effects, and antitumor therapy is facing great challenges [2]. The rise of nanomedicines optimizes the structure of antitumor drugs, improves drug retention in the tumor, and promotes antitumor therapy [3–5]. In addition, nanotechnology combined with immunotherapy, etc., to form new nanomedicines, producing synergistic antitumor effects, will become a new strategy for tumor treatment [3–5]. Currently, a variety of small-molecule immunosuppressants have been approved by the Food and Drug Administration for clinical use, but due to factors such as drug metabolism and body drug clearance, fewer molecular inhibitors reach the tumor site, which makes immunotherapy ineffective [6,7]. Therefore, more efforts should be made to develop novel nanocarriers for safer and more effective cancer immunotherapy.

In recent years, photodynamic therapy (PDT) has gained increasing attention due to its noninvasive and controllable nature [8,9]. PDT is a local tumor therapy that first delivers photosensitizers, such as protoporphyrin IX (PIX), to the tumor site and then undergoes irradiation with specific wavelengths of infrared light to generate reactive oxygen species (ROS) and cause endoplasmic reticulum stress, which ultimately leads to the immunogenic cell death (ICD) of the tumor cells, and is one of the modalities for inducing tumor immunotherapy [8,9]. PDT, due to its noninvasive nature, is often used in synergistic antitumor therapy with immunotherapy and molecularly targeted therapies under nanocarriers [10,11]. PDT can induce ICD; however, ICD-induced CD8+ T cells result in limited antitumor immunity due to immune escape from immune checkpoints such as PD-1/PD-L1 and CTLA4 on the surface of tumor cells [12]. Therefore, it is necessary to combine PDT with immunotherapy in order to further improve antitumor capacity effectively. In recent years, although studies have reported that the combination of nanocarriers and PDT can enhance the efficacy of immunotherapy [13–18], there are still some problems to be solved to fabricate more desirable nanocarrier systems. For example, because cancer cells often establish an immunosuppressive tumor microenvironment (TME), this results in effector T cells remaining ineffective at killing tumors [13,19]. In addition, small-molecule peptide nanomedicines are susceptible to degradation and rapid clearance upon entry into the blood circulatory system, which usually limits the therapeutic efficacy of peptide nanomedicines [20,21]. Therefore, there is a need to further improve the efficiency of effector T cells, prolong the circulation time of peptide nanomedicines in vivo, and improve stability.

Macitentan (MAC) is a T-cell agonist with the ability to regulate T-cell subsets to promote TME remodeling, which can stimulate CD8+ T-cell activity and promote CD8+ T-cell proliferation, thus enhancing the antitumor immunotherapy effect [22,23]. Therefore, for the first time, we combined MAC with a PD-L1-targeting peptide to coblock the immune checkpoint PD-L1/PD-L1 and enhance the effect of T-cell action. It was shown that polyethylene glycol (PEG) modification was able to evade reticuloendothelial system clearance and reduce the systemic toxicity of the drug by enriching the tumor site through enhanced permeability and retention (EPR) effects [24–28]. In addition, PEG can prolong the drug half-life so that the drug has more than sufficient time to reach the target site and increase the drug concentration locally in the tumor. Therefore, in order to improve its stability in blood circulation and protect the peptide from enzymatic degradation, we innovatively introduced PEG to combine with CVRARTR-PIX (CP) to form CPP. With this structure, CPP remains stable during blood circulation, while upon entering tumor tissues, it can detach from the PEG canopy for stronger tumor accumulation and cellular internalization [24,25].

Although a variety of nanocarriers have been used for in vivo drug delivery and a variety of peptide nanodrugs for synergistic photodynamic immunotherapy have been reported, multifunctional carriers that can deliver drugs safely and efficiently to specific sites of action have rarely been reported and have failed to achieve clinical translation and application. Therefore, for the first time, we designed a novel multifunctional peptide nanocarrier, CPP, to form a new peptide nanomedicine (CPPM) after wrapping the T-cell agonist MAC. The aim of this study is to develop a dual-activatable nanoimmunomodulator that can be used in cancer therapy, with a view that the nanomedicine can efficiently activate T cells to synergistically enhance the effect of tumor PDT and at the same time solve the problems of drug cycling stability, tumor immune escape, and insufficient immune activation and ultimately achieve safe and efficient synergistic antitumor ability.

## Materials and Methods

### Material reagents

Mouse lung epithelial cells (MLE12, BNCC337698), mouse breast cancer cells (4T1, BNCC338397), human breast cancer cells (MCF-7, BNCC100137), and mouse colon cancer cells (CT26, BNCC342605) were purchased from BeiNaChuanglian Biotechnology Co. BALB/c female mice of 4 to 6 weeks of age were purchased from Shanghai Slaughter Laboratory Animal Co. Dulbecco’s modified Eagle medium, fetal bovine serum, Hoechst 33342, and 4′,6-diamidino-2-phenylindole (DAPI) were purchased from Thermo Fisher Scientific Inc. Reactive Oxygen Detection Kit, MTT (3-(4,5-dimethylthiazol-2-yl)-2,5-diphenyltetrazolium bromide) Assay Kit, and a calcein-AM/propidium iodide (PI) double-staining kit were purchased from FANDER (Beijing) Biotechnology Co. PIX was purchased from Yuanye Biotechnology Co. MAC was purchased from MedChemExpress LLC. *N*,*N*′-Diisopropylcarbodiimide (DIC) and 4-dimethylaminopyridine (DMAP) were purchased from Beijing Huamaike Biotechnology Co. *N*-Formyldimethylamine (DMF) was purchased from Shanghai Yubo Biotechnology Co. 1-Hydroxybenzotriazole (HOBT) was purchased from Beijing Biolabs Technology Co. Piperidine (Pip) was purchased from Beijing Bailing Wei Technology Co. *O*-Benzotriazol-1-yl-tetramethyluronium (HBTU) was purchased from Shanghai Covalent Chemical Technology Co. 4-Methylmorpholine (NMM) and trifluoroacetic acid (TFA) were purchased from Shanghai Aladdin Biochemical Technology Co. Hydrazine hydrate, *N*-(9-fluorenylmethoxycarbonyloxy)succinimide (Fmoc-Osu), Wang resin, and Fmoc-Arg (Pbf)-OH were purchased from Merck. *N*,*N*-Diisopropylethylamine (DIPEA) was purchased from Shanghai Weinie Chemical Technology Co. PEG_5000_-Mal was purchased from Hangzhou Allpeptide Biotechnology Co. Anti-calreticulin (anti-CRT) and anti-high mobility group protein B1 (anti-HMGB1) were purchased from Abbott Antibodies (Shanghai) Trading Co. Alanine transaminase, blood urea nitrogen, uric acid, and aspartate aminotransferase kits were purchased from Elabscience Biotechnology Co. Other commonly used reagents were purchased from Sinopharm Chemical Reagent Co.

### Synthesis of CPPM

**Fig. 1.**
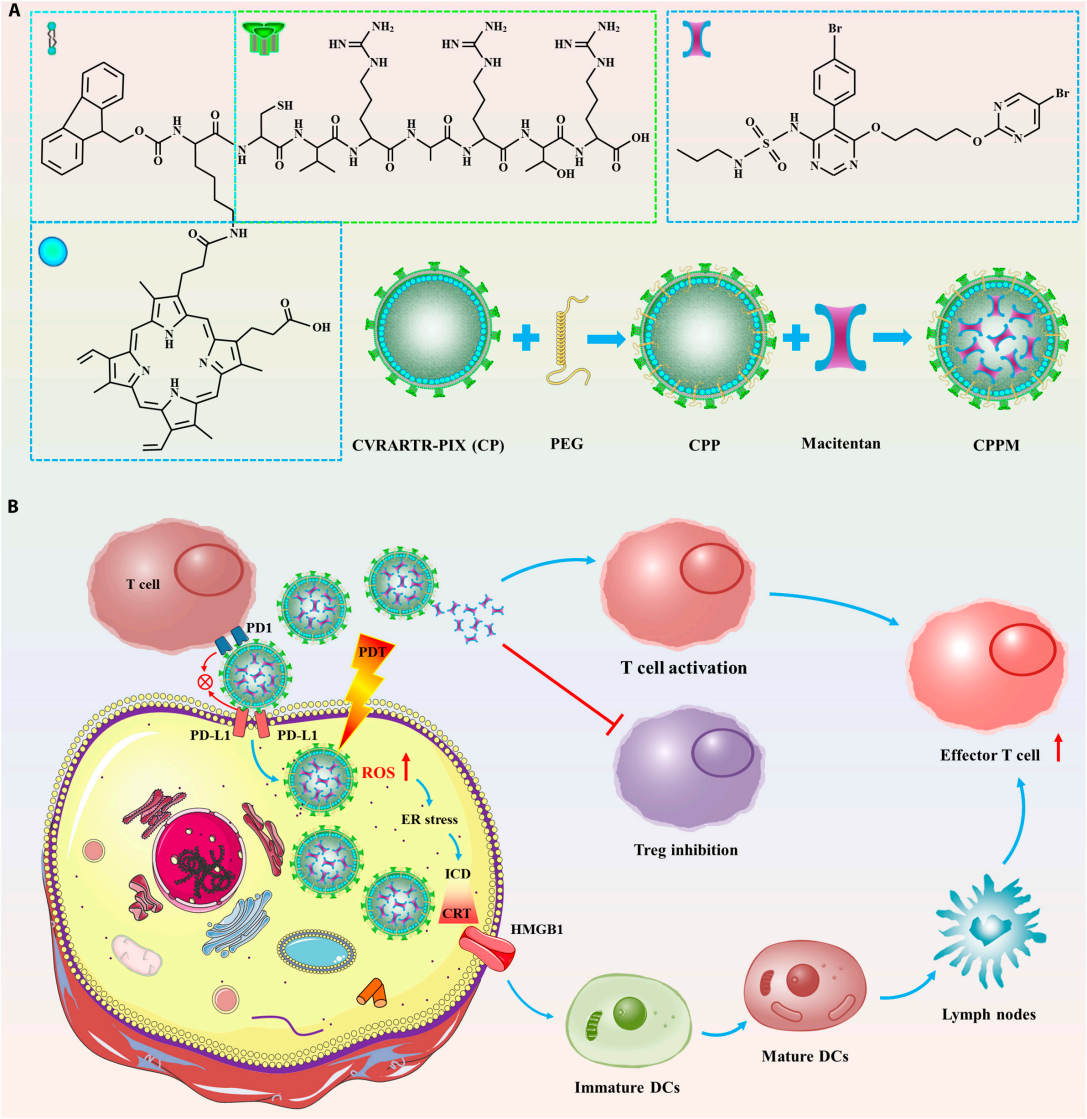
Schematic diagram of the synthesis process and action of CPPM for photodynamic therapy (PDT) and tumor immunotherapy. (A) Flowchart of CPPM synthesis. (B) Diagram of the in vivo mechanism of action of CPPM. CPPM selectively aggregates at the tumor site where PD-L1 is overexpressed, blocks the binding of PD1 on the surface of T cells to the surface of PD-L1 on the surface of the tumor cells, and revitalizes T-cell activity. Meanwhile, the T-cell agonist macitentan (MAC) can synergistically block the immune checkpoint PD-L1/PD-L1 and enhance the effect of T-cell action. Under specific light irradiation, CPPM generates reactive oxygen species (ROS), induces endoplasmic reticulum stress in tumor cells, exposes calreticulin (CRT) in the immunogenic cell death (ICD) cascade response, releases HMGB1 extracellularly, promotes the maturation of dendritic cells (DCs) and presents antigens to reach the lymph node site, activates effector T cells, and further enhances the antitumor immune response. PIX, protoporphyrin IX; PEG, polyethylene glycol; ER, endoplasmic reticulum; Treg, regulatory T cell.

Wang resin and Fmoc-Arg (Pbf)-OH were weighed and added into the reactor, and appropriate amounts of DIC, DMAP, and DCM were successively added to dissolve and react to obtain Fmoc-Arg (Pbf)-Wang resin. H_2_N-Arg (Pbf)-Wang resin was obtained by adding 20% Pip/DMF solution to Fmoc-Arg (Pbf)-Wang resin, reacting with nitrogen gas for 30 min, and washing with DMF solution. Fmoc-Thr (tBu)-OH was taken and added to H_2_N-Arg (Pbf)-Wang resin, and appropriate amounts of DMF, DIC, and HOBT were added and reacted for 30 min to obtain Fmoc-Thr (tBu)-Arg (Pbf)-Wang resin. H_2_N-Thr (tBu)-Arg (Pbf)-Wang resin was obtained by adding 20% Pip/DMF solution to Fmoc-Thr (tBu)-Arg (Pbf)-Wang resin, reacting with nitrogen gas for 30 min, and then washing with DMF solution. Repeating the above steps, Fmoc-Arg (Pbf)-OH, Fmoc-Ala-OH, Fmoc-Arg (Pbf)-OH, Fmoc-Val-OH, and Fmoc-Cys (Trt)-OH were successively added to obtain H_2_N-Cys (Trt)-Val-Arg (Pbf)-Ala-Arg (Pbf)-Thr (tBu)-Arg (Pbf)-Wang resin. Then, Dde-Lys (Fmoc)-OH, DMF, DIC, and HOBT were added for the reaction, which was washed after 30 min to obtain Dde-Lys (Fmoc)-Cys (Trt)-Val-Arg (Pbf)-Ala-Arg (Pbf)-Thr (tBu)-Arg (Pbf)-Wang resin. Then, 20% Pip/DMF solution was added and the reaction was carried out for 30 min to obtain Dde-Lys-Cys (Trt)-Val-Arg (Pbf)-Ala-Arg (Pbf)-Thr (tBu)-Arg (Pbf)-Wang resin. PIX, DMF, HBTU, and NMM were added to the above resins for reaction, respectively, and cut with the addition of 4% hydrazine hydrate/DMF solution to obtain H_2_N-Lys (PIX)-Cys (Trt)-Val-Arg (Pbf)-Ala-Arg (Pbf)-Thr (tBu)-Arg (Pbf)-Wang resin. Fmoc-Lys (PIX)-Cys (Trt)-Val-Arg (Pbf)-Ala-Arg (Pbf)-Thr (tBu)-Arg (Pbf)-Wang resin was obtained by further addition of Fmoc-Osu and reaction with DIPEA. Further addition of cutting fluid (TFA) for cutting yielded Fmoc-Lys (PIX)-Cys-Val-Arg-Ala-Arg-Thr-Arg-OH, which can be purified to yield the new photosensitizer bonding compound CVRARTR-PIX (CP) (Fig. S1). The synthesis of CP was analyzed by high-performance liquid chromatography (HPLC) and mass spectrometry (chromatographic column model: COSMOSIL 5C18-MS-II 4.6 ID*250 mm; liquid phase model: Waters 2695 Separations Module-Waters 2996 Photodiode Array Detector; test wavelength: 220.0 nm). Finally, CP was cut off from the resin with TFA/triisopropylsilane/H_2_O (95%/2.5%/2.5%), and PEG_5000_-Mal was added to assemble with CP to form C (PEG_5000_-Mal)-VRARTR-PIX (CPP). CPP was concentrated and dried in a vacuum drying oven and stored in a drying tower. The synthesized CPP was configured into a solution of 100 mg/ml and MAC into a solution of 10 mg/ml, and after mixing the 2 at a ratio of 10:3, the 2 were ultrasonicated in 1 ml of water for 10 min, and the obtained solution was dialyzed for 2 h in a 1,000-D dialysis bag, and CPP wrapped around MAC to form stable polypeptide nanomedicines (CPPM) through self-assembly effects (Fig. 1A) [13–15,24,25].

### Physical and chemical characterization tests

The synthesized nanodrugs were diluted and tested for particle size and zeta potential by a Malvern particle sizer and tested for stability over a period of 7 d. The nanodrugs were lyophilized and added to agate mortar along with potassium bromide powder, ground well, and pressed into tablets and scanned using a Fourier transform infrared spectrometer (FTIR). Electron microscopy samples were prepared by diluting the nanodrugs, and the morphology and distribution of the samples were observed by transmission electron microscopy.

### Testing of drug content and its release properties

CPP and CPPM were diluted to 1 mg/ml each. MAC was quantified by HPLC with a mobile phase A of water (0.2% TFA), a mobile phase B of acetonitrile, a flow rate of 1 ml/min, and a gradient elution of 10% to 100% B. The peak area of different concentrations of MAC was tested at 277 nm out, and the standard curve was plotted and the drug concentration was tested. PIX was quantified by ultraviolet–visible absorption spectrometry (UV–Vis), and the absorption values of different concentrations of PIX were determined at 630 nm, and the standard curve was plotted and the concentration of PIX was tested. Subsequently, the drug release profiles were determined by HPLC in release media at potential of hydrogen (pH) 5.3 and 7.4.

### CPPM targeting tumor cell PD-L1 ability and cellular uptake assay

MLE12, 4T1, MCF-7, and CT26 cells were seeded into cell culture dishes, respectively, and incubated with normal medium and medium containing interferon γ (IFN-γ) (25 ng/ml) for 48 h, respectively, and then incubated with the addition of CPPM (PIX of 6 μg/ml) for 6 h, and then the intracellular distribution of CPPM was observed under the microscope. To explore the uptake behavior of CPPM in tumor cells with high PD-L1 expression, CT26 cells were seeded into cell culture dishes, and CPPM with PIX concentrations of 6, 12, and 18 μg/ml was added, and the medium was discarded after 6 h of incubation. Alternatively, CPPM with a PIX concentration of 12 μg/ml was added and incubated for 2, 4, and 8 h, respectively. Subsequently, fresh medium containing Hoechst 33342 was added and incubated for 20 min before observation by microscopy.

### Intracellular ROS detection

The CT26 cells were incubated for 6 h with the addition of MAC, CPP, and CPPM materials, respectively, after wall culture. Subsequently, a 2,7-dichlorodihydrofluorescein diacetate probe was added to stain the cells for 30 min, and the CPP light group and CPPM light group were illuminated for 3 min, and the cells were observed and analyzed by microscopy. The experimental groupings were (the same groupings as below): blank group (blank), MAC group (MAC), CPP group (CPP), CPP light exposure group (CPP+), CPPM group (CPPM), and CPPM light exposure group (CPPM+). The concentration of PIX in CPP was 6 μg/l. The concentrations of PIX and MAC in CPPM were 6 and 6.72 μg/l, respectively.

### In vitro ^1^O_2_ assay

To further explore the ROS-producing behavior of CPPM, MAC, CPP, and CPPM were prepared into 1-ml solutions with phosphate-buffered saline, and 10 μl of reactive oxygen Singlet Oxygen Sensor Green probe was added to each set of solutions. The resulting fluorescence intensity was measured as F0 in an F-302 model fluorescence spectrophotometer (excitation wavelength of 488.0 nm, wavelength range of 500.0 to 550.0 nm), and the fluorescence intensity was measured as Ft under 630-nm laser irradiation every 15 s. The concentration of PIX in CPP was 3 μg/l. The concentrations of PIX and MAC in CPPM were 3 and 3.3 μg/l, respectively.

### Cytotoxicity test by the MTT method

CT26 cells were seeded into 96-well plates, and materials with different concentration gradients were prepared. Different materials were added into 96-well plates at 100 μl per well according to the concentration gradient, respectively. The highest concentration of PIX in CPP was 6 μg/ml. The highest concentrations of PIX and MAC in CPPM were 6 and 6.72 μg/ml, respectively. After 6 h of incubation, the CPP light group and CPPM light group were irradiated using infrared light for 3 min, and after 24 h, 20 μl of MTT reagent was added, and the absorbance was detected by an enzyme meter (570 nm) and the cell viability was calculated.

### Live/dead cell double-staining assay

MAC, CPP, and CPPM were added to CT26 cells, and the light group was incubated for 6 h followed by light for 5 min. After washing, the cells were stained with an annexin V–fluorescein isothiocyanate (FITC)/PI kit and 2 μg/ml CAM to stain the live cells and 6 μg/ml PI to stain the dead cells, and the cells were observed and analyzed by microscopy after 30 min of staining. The concentrations of PIX and MAC in CPPM were 3 and 3.3 μg/l, respectively.

### Flow cytometric apoptosis assay

After the CT26 cells were cultured on the wall, MAC, CPP, and CPPM were added and incubated for 6 h, and the light group was light-exposed for 5 min, respectively. After the cells were washed, 1× annexin V–FITC (195 μl), annexin V–FITC (5 μl), and PI staining solution (10 μl) were added, respectively, and the reaction was detected by flow cytometry after 10 to 15 min. The concentrations of PIX and MAC in CPPM were 3 mg/l and 3.3 μg/l, respectively.

### ICD immunofluorescence assay

MAC, CPP, and CPPM were added to CT26 cells, and the light group was incubated for 6 h and then irradiated with light-emitting diode light (630 nm, 29.8 mW cm^−2^) for 10 min. After continuing incubation for 4 h, paraformaldehyde (4%) and 0.1% Triton X-100 were added for fixation and permeabilization of cells, respectively. Subsequently, the cells were incubated with CRT or HMGB1 fluorescent primary antibody overnight (500:1), secondary antibody for 1 h, and DAPI for 20 min and then observed by microscopy. The PIX concentration was 6 μg/ml, and the MAC concentration was 6.72 μg/ml.

### Western blot measurement

Protein samples were added to the gel spiking wells, and the proteins were separated and transferred to polyvinylidene fluoride membranes, which were closed, and then incubated with primary (1:1,000) and secondary (1:3,000) antibodies, respectively. Enhanced chemiluminescence color development solution was added under light-avoiding conditions and photographed in a dark room. Finally, analysis was performed using the ImageJ software.

### Pharmacokinetics

Twelve healthy BALB/c female mice were randomly divided into 2 groups and injected via the tail vein with CPM (without PEG) and CPPM, respectively, with a PIX content of 1.8 mg/kg PIX. Then, blood was collected at 0, 0.5, 1, 2, 4, and 6 h after drug administration and centrifuged at 3,000 rpm for 3 min to obtain plasma samples. Absorbance was measured at 630 nm for quantitative analysis of PIX in plasma samples.

### In vivo drug distribution and imaging analysis

CT26 cells were inoculated in the axilla of BALB/c female mice to establish a mouse loaded tumor model. After the volume of the loaded tumor reached 100 mm^3^, CPPM was injected through the tail vein. The levels of PIX and MAC were 1.8 and 2.0 mg/kg, respectively. After injection for 0.5, 1, 3, and 6 h, respectively, the fluorescence distribution was examined using a small animal in vivo imager. In addition, 9 h after tail vein administration, mice were euthanized and heart, liver, spleen, lung, kidney, and tumor tissues were removed for quantitative analysis of fluorescence distribution using a fluorescence imager. After tissue homogenization, PIX in tissue samples was quantified by measuring the absorbance at 630 nm.

### Tumor growth inhibition and biosafety assays

CT26 cells were inoculated on the back of BALB/c female mice to construct a subcutaneous tumor model (Fig. S2A). The groups were treated as follows (the same groupings as below): MAC (2.0 mg/kg), CPP (PIX content of 1.8 mg/kg), and CPPM (PIX and MAC contents of 1.8 and 2.0 mg/kg, respectively) were injected separately through the tail vein, and the light group was irradiated using a laser for 6 h after drug administration for 10 min (intensity of 50% and power of 0.68 W). The mice were treated every 2 d for a total of 5 treatments, and tumor volume and body weight were measured every other day. On day 18, the mice were executed by cervical dislocation, and their blood was removed and dissected. The spleens of the mice were weighed, and the mouse tumor tissues were stained for Ki67, terminal deoxynucleotidyl transferase dUTP nick end labeling (TUNEL), and CD3/CD8. Different concentrations of CPPM solutions were prepared with saline; 100 μl of CPPM solution was added into 2 ml of mouse blood and mixed well and incubated in a 37 °C water bath for 1 h. After centrifugation, 100 μl of the supernatant was taken into a 96-well plate, and the absorbance value was measured by an enzyme-labeling instrument at a wavelength of 540 nm and the hemolysis rate was calculated. In addition, blood biochemistry and blood routine indexes were analyzed [29,30], and hematoxylin–eosin (H&E) staining was performed on tumor tissues and major organs.

### Intratumor immune cell analysis assay

CT26 cells were inoculated in the back of BALB/c female mice after the tumor volume reached 100 mm^3^. The treatment was performed every 2 d for 3 treatments (Fig. S2B). After the completion of treatment, the subcutaneous tumors of mice were removed, and the tumor tissues were clipped and digested using tissue digestive solution for 1 h. The digested murky suspension was collected, and the cells were resuspended after washing with phosphate-buffered saline. Dyes corresponding to regulatory T (Treg) cells, CD3+ CD4+ CD8+ T cells, and dendritic cells (DCs) were added, respectively, and the corresponding proportions of the stained cells were detected by flow cytometry. The spleens of mice in each group were removed, ground and added to erythrocyte lysate, and lysed for 20 min to wash and collect spleen cells; the dye corresponding to CD3+ CD4+ CD8+ T cells was added; and the proportion of CD3+ CD4+ CD8+ T cells was detected by flow cytometry.

### In vivo antitumor metastasis studies

The mouse lung metastasis model was established by inoculating CT26 cells on the back of BALB/c female mice and injecting 2 × 10^6^ CT26 cells labeled using green fluorescent protein through the tail vein when the tumor volume of the loaded mice reached 100 mm^3^ (Fig. S2C). The groups were treated in the same manner as those in the immune response study. The mice were executed after 10 d of treatment, and the lung tissues of the mice were excised and photographed, and the metastatic lung nodules were quantified and H&E-stained for further examination of the extent of lung metastasis.

### Statistical analyses

The GraphPad Prism 8.0.2 software was used for statistical analysis, and data are expressed as mean ± SD. Significance levels are expressed as *P* < 0.05 (*), *P* < 0.01 (**), and *P* < 0.001 (***).

## Results

### Physical and chemical characterization tests

The HPLC plot of CP shows that the purity of the target product was 97.78% during the run from 40% to 100% in 20 min (Fig. S3). The target product was analyzed by mass spectrometry (Fig. S4), and the value of [M + 4H] was 440.12, that of [M + 3H] was 586.46, and that of [M + 2H] was 879.45, and the calculations gave the molecular weights of the target product as 1,756.48, 1,756.38, and 1,756.9, respectively, whereas the theoretical molecular weight of the target product was 1,756.11; the molecular weight size was within the error, indicating that the target product CP was synthesized successfully.

**Fig. 2.**
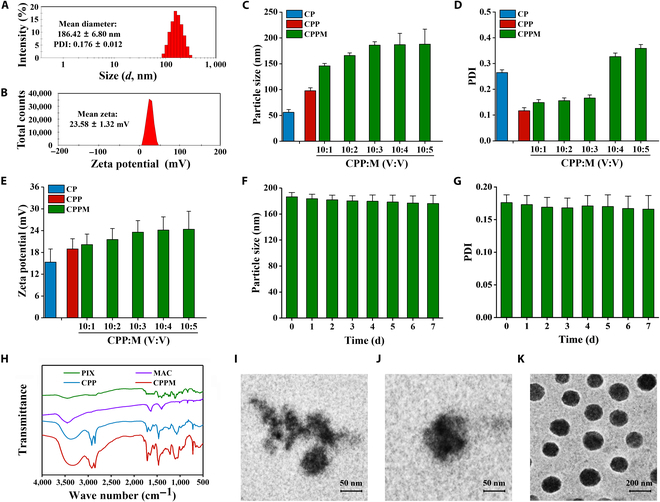
Physicochemical characterization tests of CPPM. (A) Particle size distribution of CPPM. (B) Zeta potential distribution of CPPM. (C) Particle sizes of CP, CPP, and CPPM with different drug contents. (D) Polydispersity indexes (PDIs) of CP, CPP, and CPPM with different drug contents. (E) Zeta potentials of CP, CPP, and CPPM with different drug contents. (F) CPPM particle sizes within 7 d (for CPP:M of 10:3). (G) CPPM PDIs within 7 d (for CPP:M is 10:3). (H) Fourier transform infrared spectrometer (FTIR) scanning curves of the nanodrugs. (I) TEM morphology of CP. (J) TEM morphology of CPP. (K) Transmission electron microscopy (TEM) morphology of CPPM.

The particle size and potential of CPPM were (186.42 ± 6.80) nm and (23.58 ± 1.32) mV, respectively, and the polydispersity index (PDI) in solution was (0.176 ± 0.012) (Fig. 2A and B). After the introduction of PEG into CP, the particle size of CPP increased, and with the increase in MAC content, the particle size gradually increased, and the optimal wrapping state was reached when the ratio of CPP to MAC was 10:3 (Fig. 2C). In addition, the PDI of CP was larger, and after the introduction of PEG, the PDI of CPP decreased, and with the increase in MAC content, the PDI increased slowly, and when the ratio of CPP to MAC was more than 10:3, the PDI increased (Fig. 2D), and the nanomedicine always remained positively charged (Fig. 2E). Therefore, the ratio of 10:3 was chosen for the subsequent study, which not only could encapsulate more MAC but also had the advantages of good dispersion and uniform particle size distribution. Both the particle size and PDI values of CPPM did not change substantially within 7 d, indicating good stability (Fig. 2F and G). Subsequently, the FTIR spectra of PIX, MAC, CPP, and CPPM were scanned (Fig. 2H), and the characteristic peaks of PIX could be observed in CPP, and those of CPP and MAC in CPPM, indicating the successful construction of the photosensitizer-containing peptide nanomedicine. Transmission electron microscopy showed that both CP and CPP had irregular morphology and agglomeration of CP occurred, and CPPM after encapsulation of the MAC drug was a nanovesicle with regular morphology (Fig. 2I to K).

In order to study the contents of PIX and MAC in nanomedicines, PIX was quantitatively tested by UV–Vis, and the standard curve of PIX was plotted (Fig. S5A), and the concentration of PIX in CPP was finally measured to be 120.91 μg/ml, and the concentration of PIX in CPPM was 96.37 μg/ml (Fig. S6). The loading efficiency of PIX in CPP was 12.09%, and that of PIX in CPPM was 9.64% (Fig. S7). MAC was quantitatively tested by HPLC, and the standard curve was plotted (Fig. S5B), and the final concentration of MAC in CPPM was measured to be 103.53 μg/ml (Fig. S6). The loading efficiency of MAC in CPPM was 10.35% (Fig. S7). Subsequently, we evaluated the drug release behavior of CPPM at different pH levels and found that MAC was released more rapidly under acidic conditions (Fig. S8), suggesting that CPPM prefers to release drugs in the acidic TME, enabling effective delivery of MAC and thus enhancing antitumor immunotherapy.

### Targeted uptake of CPPM by tumor cells and photodynamic performance testing

**Fig. 3.**
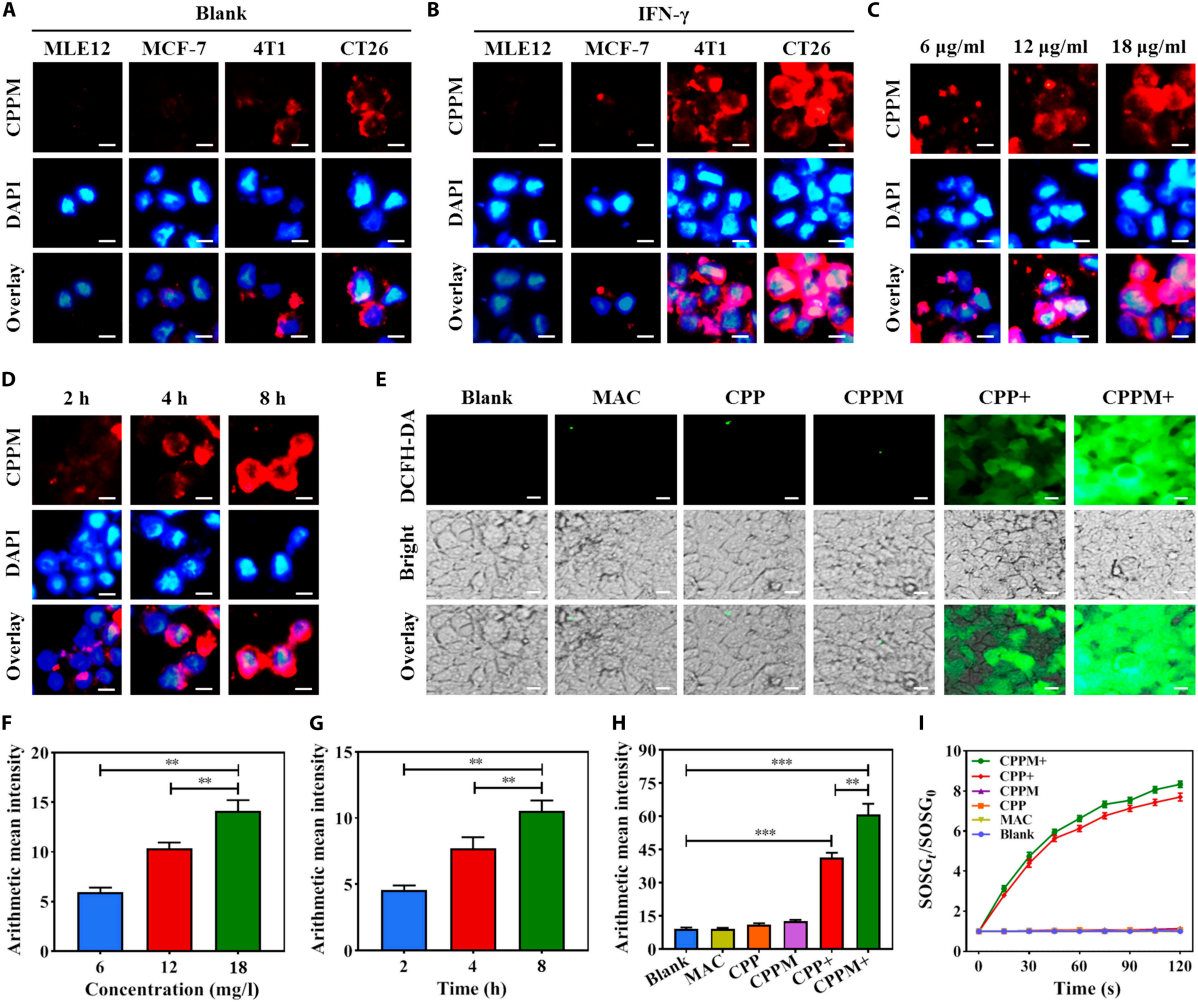
Targeted uptake of CPPM by tumor cells and its detection of ROS generation inside and outside the cell. (A) Targeted attachment of CPPM in cell lines with different PD-L1 expression levels (scale bar: 10 μm). (B) Targeted attachment of CPPM in cell lines with different PD-L1 expression levels after addition of interferon γ (IFN-γ; scale bar: 10 μm). (C) CT26 uptake behavior for different concentrations of CPPM at the same time (scale bar: 10 μm). (D) CT26 uptake behavior for the same concentration of CPPM at different times (scale bar: 10 μm). (E) Observations after endocytosis of MAC, CPP, and CPPM in the presence or absence of light (scale bar: 10 μm). (F) Average fluorescence intensity of CPPM uptake by CT26 in concentration gradient experiments (*n* = 3). (G) Average fluorescence intensity of CT26 uptake of CPPM in time gradient experiments (*n* = 3). (H) Fluorescence intensity analyzed using the 2,7-dichlorodihydrofluorescein diacetate (DCFH-DA) fluorescent green reagent as a ROS probe under illuminated or unilluminated conditions (*n* = 3). (I) The ^1^O_2_ generation capacity of MAC, CPP, and CPPM was detected using Singlet Oxygen Sensor Green (SOSG) as a ^1^O_2_ probe in the presence or absence of light. DAPI, 4′,6-diamidino-2-phenylindole.

In the normal medium incubation group, CPPM-targeted attachment was higher on the cell membrane surface of CT26 and 4T1 cells with high PD-L1 expression than on that of normal cells MLE12, while no CPPM-targeted attachment was seen on the surface of MCF-7 with low PD-L1 expression (Fig. 3A). After the medium was treated with IFN-γ, the targeting effect of CPPM on the cell membranes of CT26 and 4T1 was more pronounced as IFN-γ could stimulate to increase the level of PD-L1 expression on the surface of the cancer cells, suggesting that CPPM could target PD-L1 on the membranes of tumor cells (Fig. 3B). For MLE12 and MCF-7 cells, which did not express or lowly expressed PD-L1, no obvious expression of CPPM was seen on the cell membrane after IFN-γ incubation, indicating that the level of PD-L1 expression on the membranes of these 2 types of cells was extremely low and was not affected by IFN-γ. The fluorescence intensity of PIX in CT26 cells by CPPM showed that the higher the concentration of CPPM, the stronger the fluorescence intensity of PIX in CT26 at the same time (Fig. 3C and F). The longer the CT26 uptake time at the same concentration, the stronger the fluorescence intensity of PIX in CT26 (Fig. 3D and G). This indicated that the behavior of CPPM uptake by CT26 cells was concentration dependent and time dependent. The results of photodynamic experiments showed that no green fluorescence was seen in the dark treatment of the CPP and CPPM groups, indicating that the CPP and CPPM groups did not produce ROS and were not dark toxic under no-light conditions. Both the CPP and CPPM light groups showed green fluorescence, suggesting that CT26 cells could produce ROS under light conditions and that the CPPM was more capable of producing ROS (Fig. 3E and H), which might be related to the relatively large specific surface area of CPPM. In addition, both CPP and CPPM could produce ^1^O_2_ under light conditions, and the fluorescence was enhanced with the increase in time (Fig. 3I), which indicated that both CPP and CPPM had good photodynamic properties and CPPM was more capable of generating ROS under light conditions, which could better exert its antitumor therapeutic effect.

### Studies on the antitumor effects of CPPM in vitro

The antitumor effects of CPPM were evaluated by the MTT method, and no significant toxic effects were shown in the MAC, CPP, and CPPM groups (Fig. S9A), while CPP+ and CPPM+ showed stronger cytotoxicity, which was enhanced with the concentration of the administered drug in a concentration-dependent manner (Fig. S9B). This indicated that both CPP and CPPM were able to kill tumors in the presence of light, and the ability of CPPM to kill tumors was stronger. Subsequently, the flow apoptosis technique was used to detect CT26 cell apoptosis, and the MAC, CPP, and CPPM groups had lower apoptosis rates, and apoptosis was significantly higher in the CPP+ and CPPM+ groups (Fig. S9C and D). This indicated that MAC, CPP, and CPPM had very weak dark toxicity and high biosafety. In the presence of light, CPP and CPPM had strong phototoxicity, and CPPM had stronger phototoxicity. Using the live/dead double-staining method to observe the cell state directly under the microscope, both CPP and CPPM had cell death after PDT, and CPPM had a higher cell death rate after PDT (Fig. S9E). The above results indicated that CPPM-mediated PDT treatment was more effective and showed strong antitumor ability.

### Study of the PDT mechanism of CPPM

It was found by immunofluorescence experiments that both CPP and CPPM were able to increase the expression of CRT on the cell membrane under PDT (Fig. S10A and B), while HMGB1 was significantly attenuated in tumor cells (Fig. S10C and D). This indicated that ICD was induced by PDT treatment with CPP and CPPM, and the effect of CPPM was more obvious. The results of protein expression assay showed that CRT protein expression was elevated in the CPP and CPPM groups after PDT, and the elevated CRT protein expression was more obvious in the CPPM group (Fig. S10E and F). HMGB1 protein expression was decreased in the CPP and CPPM groups after PDT, and HMGB1 protein expression was decreased more obviously in the CPPM group (Fig. S10E and G). The protein expression results were consistent with the CRT and HMGB1 immunofluorescence of cells; CRT cell membrane exposure, HMGB1 extracellular release, and elevated CRT protein and reduced HMGB1 protein all confirmed the induction of ICD by PDT.

### Pharmacokinetics

The plasma concentration versus time curves after injection of CPM or CPPM showed (Fig. S11) that the pharmacokinetic profiles of both CPM and CPPM conformed to the one-compartment model. The plasma half-lives (*t*_1/2_) of CPM and CPPM were 53 and 119 min, respectively, compared with longer half-life for CPPM, which was 2.25 times longer than that of CPM. This showed that PEGylation of the nanocarriers could significantly prolong their blood circulation time.

### In vivo antitumor efficacy and biosafety evaluation of CPPM

**Fig. 4.**
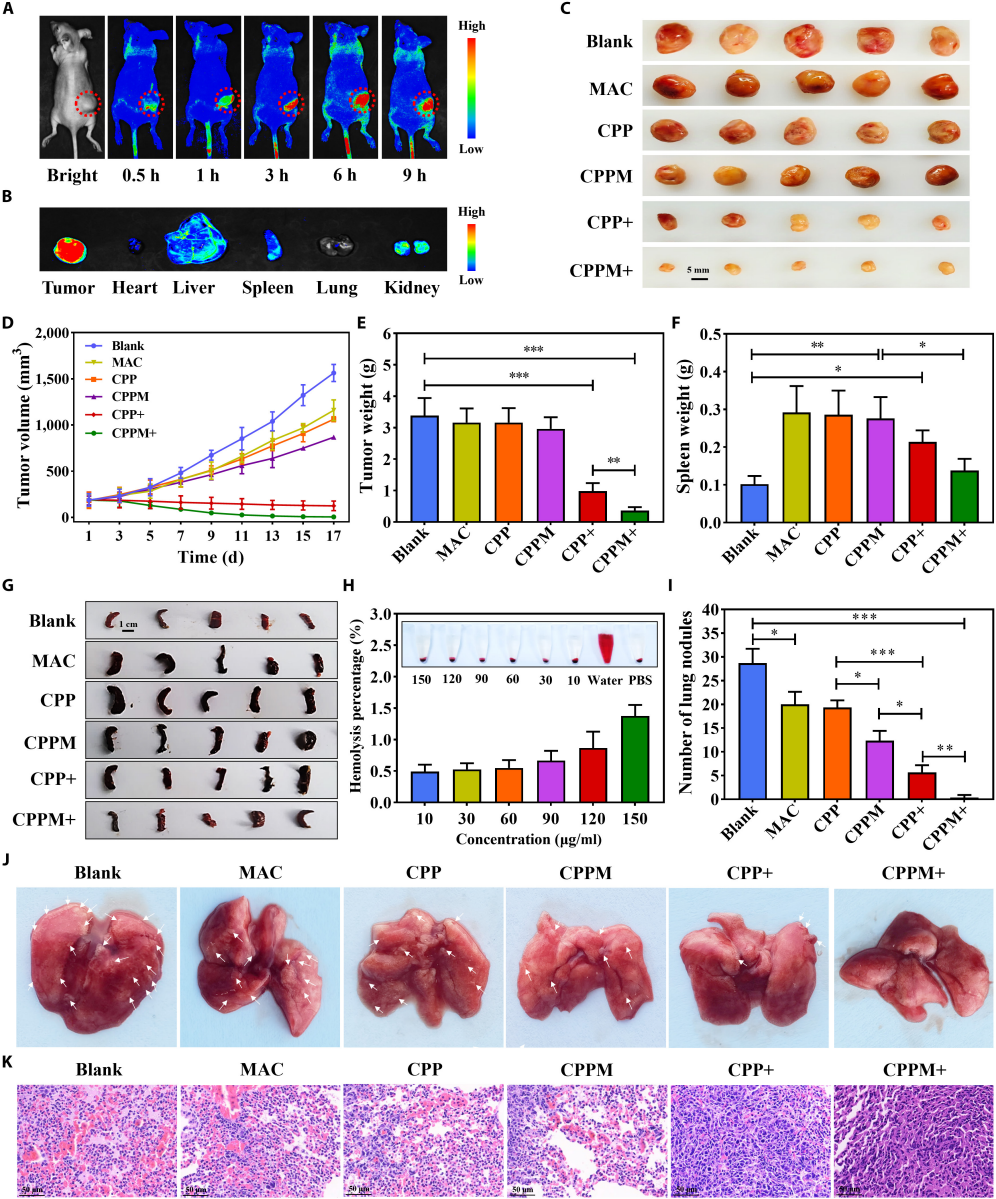
Evaluation of the in vivo antitumor growth and metastasis efficacy of CPPM. (A) Biodistribution of CPPM analyzed by real-time fluorescence imaging after tail vein injection of CPPM in mice. (B) In vitro fluorescence imaging images of heart, liver, spleen, lung, kidney, and tumor. (C) Images of tumor formation in various groups of mice. (D) Comparison of tumor volume in each group of mice (*n* = 5). (E) Comparison of tumor weight in each group of mice (*n* = 5). (F) Comparison of the average weight of the spleen in each group of mice (*n* = 5). (G) Images of isolated spleens of mice in each group. (H) Hemolysis of different concentrations of CPPM and hemolysis rate statistics (*n* = 3). (I) Statistics on the number of lung nodules (*n* = 3). (J) Ex vivo imaging images of free lung tissue. (K) Microscopic observation of lung tumor metastasis after hematoxylin–eosin (H&E) staining. PBS, phosphate-buffered saline.

Biodistribution experiments showed that CPPM preferentially aggregated at the tumor site, with increasing drug retention in the tumor with time, and after 6 h, drug retention in the tumor site began to decrease (Fig. 4A). Tumors and organs were removed from the mice after 9 h, and it was found that fluorescence remained the strongest at the tumor site and that the drug could be metabolized by the liver or kidneys (Fig. 4B). The data from the quantitative analysis of the tissue samples were consistent with the results of the fluorescent images, with a 23.8% accumulation rate of the drug in tumor tissues after 6 h of injection (Fig. S12). In the in vivo antitumor experiments, the comparative results of tumor size (Fig. 4C), tumor volume (Fig. 4D), and tumor weight (Fig. 4E) in mice all showed that the MAC, CPP, and CPPM groups exhibited low dark toxicity and low antitumor properties, whereas the CPP+ and CPPM+ groups were able to inhibit tumor growth, and the CPPM+ group demonstrated a more potent antitumor effect. This indicates that CPPM combined with PDT has superior tumor efficacy. Tumor tissue staining revealed that the CPPM+ group had the strongest TUNEL signal and the weakest Ki67 signal (Fig. S13A and B). This indicated that PDT of CPPM could inhibit tumor growth and promote tumor cell apoptosis. In addition, there was no significant difference in the comparison of mouse body weight in each group of mice (Fig. S14), and the hemolysis rate of different concentrations of CPPM was less than 2.0% (Fig. 4H), indicating that CPPM has good biocompatibility. Blood was collected for biochemical and hematological tests, and no obvious abnormalities were found in the liver function indexes (Fig. S15A and B) and kidney function indexes (Fig. S15C and D) of mice in all groups, indicating that CPPM has little effect on liver or kidney function. The results of routine blood tests showed that all groups had an inflammatory response (Table), but the indicators in the CPPM+ group were relatively better, which implied that the inflammation was improved. In the routine blood tests of the CPPM+ group, most of the other indicators did not show abnormalities, which indicated that CPPM was more compatible with blood. In addition, the results of the H&E staining of the major organs of the mice showed that the heart, liver, spleen, lungs, and kidneys did not show obvious morphological abnormalities (Fig. S16). In conclusion, CPPM has high biocompatibility and low toxicity, with minimal toxic effects on normal tissues or organs.

**Table.** Blood routine examination (mean ± SD)

Indicators	Blank	MAC	CPP	CPPM	CPP+	CPPM+	Reference
WBC (10^9^/l)	214.80 ± 69.67	132.48 ± 37.09	58.26 ± 13.14	107.18 ± 45.11	23.68 ± 8.90	15.01 ± 8.88	0.80–6.80
HGB (g/l)	131.25 ± 6.98	115.11 ± 15.08	128.72 ± 10.14	99.71 ± 15.62	96.79 ± 24.05	141.16 ± 12.47	110.00–143.00
HCT (g/l)	38.13 ± 2.64	37.42 ± 2.73	40.96 ± 2.88	32.65 ± 3.60	45.45 ± 4.27	37.78 ± 2.66	34.60–44.60
Gran (10^9^/l)	190.87 ± 64.23	116.23 ± 32.29	10.46 ± 3.67	75.70 ± 21.51	7.74 ± 3.52	3.86 ± 1.28	0.10–1.80
RBC (10^12^/l)	7.66 ± 0.49	7.82 ± 0.54	8.19 ± 0.46	6.31 ± 0.72	8.97 ± 0.97	7.65 ± 1.04	6.36–9.42
Lymph (10^9^/l)	51.38 ± 21.07	24.74 ± 5.26	42.52 ± 10.65	27.12 ± 6.74	15.32 ± 6.86	6.34 ± 2.26	0.70–5.70
Mon (10^9^/l)	11.05 ± 3.25	5.54 ± 1.77	3.09 ± 0.98	5.85 ± 2.09	1.03 ± 0.33	0.82 ± 0.31	0.00–0.30
RDW (%)	15.77 ± 0.47	14.91 ± 0.48	14.24 ± 0.37	15.27 ± 0.37	13.92 ± 0.54	14.39 ± 0.38	13.00–17.00
MCH (pg)	18.39 ± 1.19	16.61 ± 1.12	15.31 ± 0.12	15.66 ± 0.68	15.61 ± 0.25	14.93 ± 0.37	15.80–19.00
MCHC (g/l)	371.50 ± 39.37	318.78 ± 20.68	312.96 ± 5.94	320.10 ± 10.60	315.22 ± 13.18	312.39 ± 7.85	302.00–353.00
HCV (fl)	51.40 ± 2.19	52.75 ± 2.51	52.75 ± 2.05	51.09 ± 2.06	53.22 ± 3.12	48.27 ± 0.93	48.20–58.30
PCT (%)	0.27 ± 0.09	0.21 ± 0.06	0.19 ± 0.05	0.12 ± 0.04	0.12 ± 0.05	0.12 ± 0.06	ND
PDW	17.63 ± 0.85	17.89 ± 0.54	18.08 ± 0.46	17.45 ± 0.24	17.66 ± 0.58	17.91 ± 0.84	ND

WBC, white blood cells; HGB, hemoglobin; HCT, hematocrit; Gran, granulocytes; RBC, red blood cells; Lymph, lymphocytes; Mon, monocytes; RDW, red cell distribution width; MCH, mean corpuscular hemoglobin; MCHC, mean corpuscular hemoglobin concentration; HCV, hepatitis C virus; PCT, plateletcrit; PDW, platelet distribution width; ND, no data

### Immune cell analysis

**Fig. 5.**
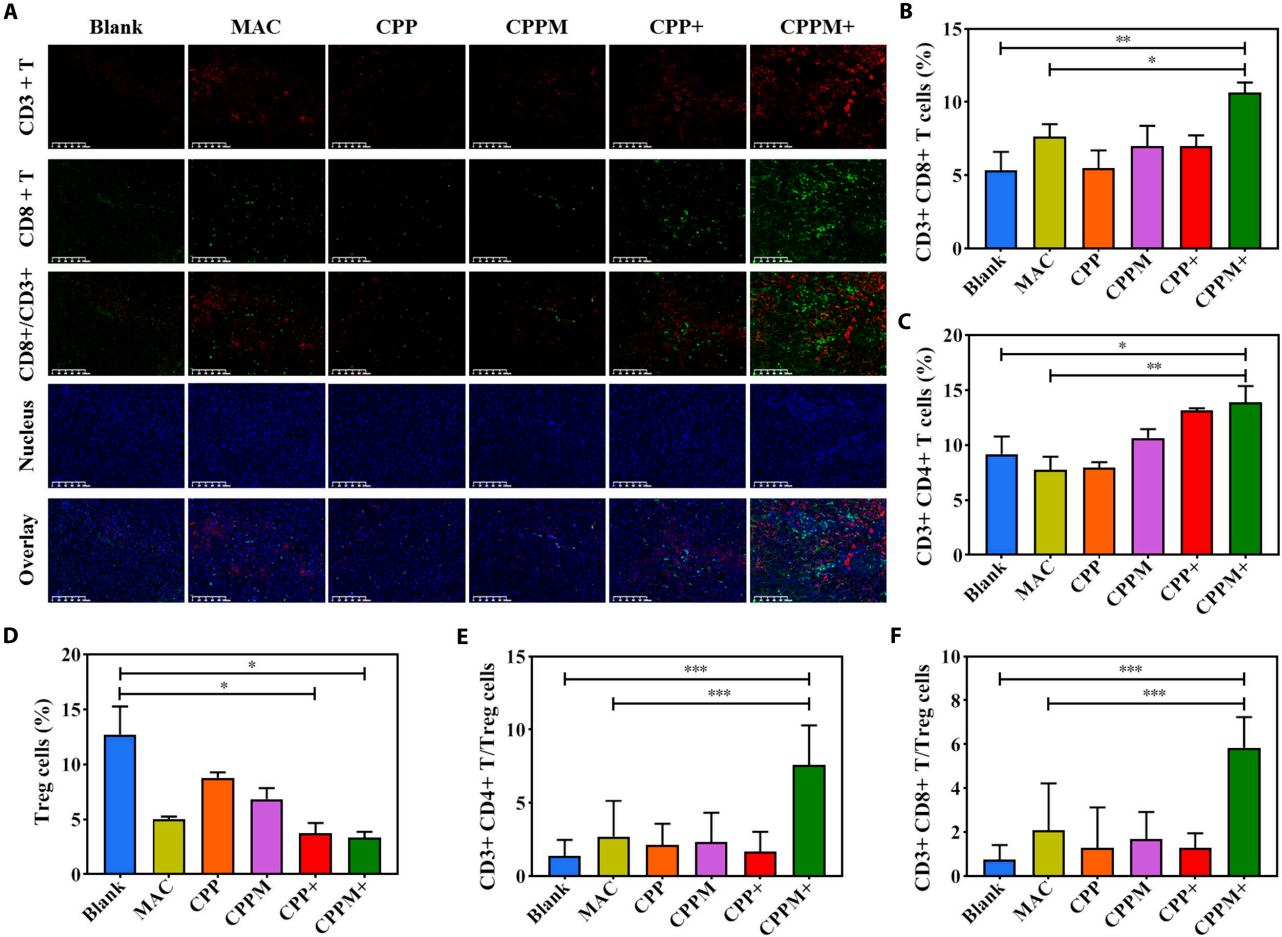
Intratumor immune cell analysis. (A) Tumor tissue sections subjected to immunofluorescence staining results for CD3+ CD8+ T cells (scale bar: 100 μm). (B) Percentage of CD3+ CD8+ T cells within the tumor analyzed by flow cytometry (*n* = 3). (C) Flow cytometric analysis of the percentage of CD3+ CD4+ T cells within the tumor (*n* = 3). (D) Flow cytometric analysis of the percentage of Treg cells within the tumor (*n* = 3). (E) CD3+ CD4+ T cells versus Treg cell percentage values (*n* = 3). (F) CD3+ CD8+ T cells versus Treg cell percentage values (*n* = 3).

After immunotherapy, it was observed that the CPPM+ group significantly ameliorated splenomegaly in mice (Fig. 4F and G), which further supported the enhancement of systemic immune response. Tumor tissues were taken for immunofluorescence staining of CD3+ CD8+ T cells (Fig. 5A), and the MAC group was able to increase the level of CD3+ CD8+ T cells in the tumors, but fewer immune cells were activated, and the tumor immunotherapy capacity was low. The CD3+ CD8+ T cells in the CPPM+ group were significantly higher in number than those in the MAC group, and more immune cells were induced than those in the CPP+ group. This indicated that CPPM combined with PDT was more able to stimulate CD3+ CD8+ T cells and enhance the antitumor immunotherapy ability. From flow cytometric analysis, the highest percentage of CD3+ CD8+ T cells and CD3+ CD4+ T cells were found in CPPM+ group (Fig. 5B and C). Tumor tissues in the CPPM+ group had the lowest percentage of CD3+ CD4+ Foxp3+ T cells (Fig. 5D), and the ratios of CD3+ CD8+ T cells and CD3+ CD4+ T cells to CD3+ CD4+ Foxp3+ T cells were up to 5-fold in the CPPM+ group (Fig. 5E and F). These results suggest that PDT of CPPM can improve the immunosuppressive TME while promoting antitumor immunotherapy.

### Evaluation of the antitumor metastasis efficacy of CPPM in vivo

To further confirm the ability of nanomedicines to synergistically enhance the efficacy of photodynamic immunotherapy, we conducted in vivo antitumor metastasis studies. The results showed that lung metastasis obviously occurred in the blank, MAC, CPP, and CPPM groups; a few lung metastases were visible in the CPP+ group; and no obvious lung metastases were seen in the CPPM+ group, which indicated that the PDT of CPPM was more resistant to tumor metastasis (Fig. 4I and J). In addition, the microscopic observation results of the H&E staining of lung tissue sections were consistent with the distribution of lung metastases (Fig. 4K). These results indicated that CPPM could inhibit tumor metastasis more efficiently under PDT.

## Discussion

In this study, we developed a dual-activatable nanoimmunomodulator that can be used in cancer therapy, which can efficiently activate T cells to synergistically enhance the effect of tumor PDT and at the same time address the problems of drug cycling stability, tumor immune escape, and insufficient immune activation, and exhibit highly efficient antitumor capabilities without causing any adverse side effects. This photodynamic immune synergistic therapeutic agent may provide an enhanced therapeutic strategy with multiple synergistic mechanisms for the treatment of advanced cancers. Meanwhile, this study provides a new strategy for developing the design of combination regimens of immune-combination therapies against tumors. In addition, other kinds of photosensitizers and T-cell agonists instead of PIX and MAC can be encapsulated into nanoparticles to achieve better tumor specificity and therapeutic efficacy.

Effective prevention and treatment of tumors is the key to improving patients’ quality of life and prolonging survival cycles [31,32]. Immune checkpoint blockade has become a means of clinical tumor treatment, which can kill abnormally proliferating tumor cells in the organism by boosting self-protective immune response and reviving effector T cells [33,34]. However, due to the loss of drug metabolism in the body, the number of immune checkpoint inhibitors that can reach the tumor site to play a role is limited, and at the same time, the immunosuppressive microenvironment in the tumor prevents the immune checkpoint blockade therapy from providing positive and long-lasting therapeutic effects [35,36]. Immunotherapy is a new direction in tumor treatment, and immune checkpoint blockade is the main target of tumor immunotherapy, while PD1/PD-L1 immune checkpoint blockade is a hot spot in immunotherapy research [37,38]. PD1/PD-L1 small-molecule immunosuppressants have been widely used in the clinic, and the role of immunosuppressant antitumor therapy is greatly reduced due to the influence of the route of administration, body metabolism, pharmacokinetics, and other factors [39,40]. Tumor-targeted therapy can solve the above problems; in particular, immune-checkpoint-targeted therapy can improve the efficiency of tumor immunity [41,42]. Tumor-targeting peptides are accepted by most antitumor strategies due to their high biocompatibility, low toxicity, and high bioavailability. With the progress of nanomedicine, tumor-targeting peptides are becoming more and more popular in antitumor therapy. Meanwhile, tumor-targeting peptides can also be used as nanocarriers loaded or coupled with antitumor drugs to form new nanomedicines, which can perform multiple antitumor functions at the same time and achieve synergistic antitumor effects [8–15].

The PD-L1 tumor-targeting peptide sequence CVRARTR in this study is a functional peptide structure and a nanocarrier, which can target competitively block PD1/PD-L1 binding, revitalize effector-T-cell activity, and have antitumor immunotherapy effects [8–12]. However, the antitumor effect of a single tumor-targeting gong peptide is far from the level of curing tumors and generally needs to be combined with other therapeutic means in order to work better [43]. For this reason, PD-L1 tumor-targeting peptides work synergistically with other treatments against tumors, often with unexpected results [44]. PDT is a localized tumor treatment modality, which destroys local tumors through light-activated ROS, leading to tumor necrosis. At the same time, PDT can induce ICD cascade antitumor immune responses, thereby increasing tumor sensitivity to immunotherapy [45,46]. The study showed that Lee et al. [47] developed a positively charged, amphiphilic anti-PD-L1 peptide nanomedicine with strong cellular and tissue permeability, capable of generating ROS and inducing ICD under laser conditions while modulating the tumor’s microenvironment, leading to antitumor immunotherapy. Zheng et al. [48] reported a drug-loaded microbubble delivery system with enhanced effects on PD-L1 blockade immunotherapy and ultimately antitumor immunotherapeutic effects. The present study has some similarity with their research material design, but the material structure and design concept are completely different. Specifically, in this study, the PD-L1 tumor-targeting peptide was first utilized to form CP by bonding with the photosensitizer PIX, and in order to improve the cyclic stability and protect the peptide from enzymatic degradation, we innovatively introduced PEG, which was assembled with CP to form CPP. The results showed that the introduction of PEG not only improved stability but also prolonged the half-life, thereby increasing the blood circulation time. CPP has both PD-L1 tumor targeting and can carry photosensitizers required for PDT and can also be used as a nanocarrier to encapsulate antitumor drugs. In order to improve antitumor immune function, massive activation of effector T cells is required to improve the tumor immunosuppressive microenvironment. Therefore, we used MAC as a drug model for the first time to both reverse the immunosuppressive TME and activate effector T cells to amplify the antitumor immune efficacy [22,23]. The results showed that CPPM could be safely and stably targeted to the tumor site for a long time through the EPR effect, specifically bind to PD-L1 on the tumor surface, increase the accumulation of the drug in the tumor cells, enhance the PDT to generate ROS, induce endoplasmic reticulum stress in the tumor cells, and combine with MAC to revitalize the activity of the T cells and promote the proliferation of the T cells, which would enhance the antitumor immune ability and ultimately kill cancer cells (Fig. 1B).

The results of this study indicate that CPPM can amplify the ability of nanomedicine to target tumors through the EPR effect, promote the attachment and collection of CPPM at the tumor site, and enhance the local tumor-killing effect of PDT at the tumor site and, at the same time, be able to promote the exposure of CRT at the cell membrane and the extracellular release of HMGB1 and induce cascading immune responses in ICDs (Fig. 1B). In the ICD cascade, CRT can release “eat-me” signals on tumor cell membranes, recruit immature DCs to phagocytose tumor cell fragments, promote DCs to mature, and present antigens to reach lymph nodes to activate effector T cells (CD3+ CD8+ T cells), further enhancing the antitumor immune response [49]. Meanwhile, CPPM can bind PD-L1 competitively with PD-1 and revitalize effector T cells. In addition, MAC released by CPPM in tumor cells can promote TME remodeling by regulating T-cell subsets, reverse immunosuppressive TME, activate CD3+ CD8+ T cells, and exert efficient antitumor ability [22,23]. We constructed a CT26 tumor-loaded tumor model subcutaneously in the hind legs of mice. The percentage of Treg cells in the CPPM group was significantly higher than that in other groups as seen by immune cell analysis, indicating that PM could reduce regulatory T lymphocytes and increase the proportion of CD3+ CD4+ CD8+ T cells in tumors. Regulating T-cell subsets through MAC promotes TME remodeling, reduces Treg cell recruitment, activates effector T cells, safely and effectively inhibits tumor cell value addition and metastasis, induces ICD through PDT, presents antigens, and activates immune responses. This indicates that CPPM has a synergistic and long-lasting antitumor effect through PDT activation of immunity with PD-L1 tumor targeting. This photodynamic immune synergistic therapeutic agent may provide an enhanced therapeutic strategy with multiple synergistic mechanisms for the treatment of advanced cancers. It is believed that in the near future, it can be applied to cancer patients through biotransformation to provide a more superior therapeutic strategy for the treatment of cancer patients.

## Ethical Approval

All experimental procedures were carried out according to the Guide for the Care and Use of Laboratory Animals and was approved by the Institutional Animal Care and Use Committee (No.: WDRM-20240901C). The study was carried out in compliance with the ARRIVE guidelines.

## Data Availability

All data generated or analyzed during this study are included in this published article (and its Supplementary Materials file).

## References

[1] Bray F, Laversanne M, Sung H, Ferlay J, Siegel RL, Soerjomataram I, Jemal A. Global cancer statistics 2022: GLOBOCAN estimates of incidence and mortality worldwide for 36 cancers in 185 countries. CA Cancer J Clin. 2024;74(3):229–263. 38572751 10.3322/caac.21834

[2] Zhong L, Li Y, Xiong L, Wang W, Wu M, Yuan T, Yang W, Tian C, Miao Z, Wang T, et al. Small molecules in targeted cancer therapy: Advances, challenges, and future perspectives. Signal Transduct Target Ther. 2021;6(1):201. 34054126 10.1038/s41392-021-00572-w PMC8165101

[3] Wu Y, Li Y, Yan N, Huang J, Li X, Zhang K, Lu Z, Qiu Z, Cheng H. Nuclear-targeted chimeric peptide nanorods to amplify innate anti-tumor immunity through localized DNA damage and STING activation. J Control Release. 2024;S0168-3659(24):00229–00223. 10.1016/j.jconrel.2024.04.008 38580138

[4] Chae SY, Shin H, Woo J, Kang S, Lee SM, Min DH. Metabolic modulation of kynurenine based on kynureninase-loaded nanoparticle depot overcomes tumor immune evasion in cancer immunotherapy. ACS Appl Mater Interfaces. 2024;16(15):18490–18502. 38573937 10.1021/acsami.4c00513

[5] Huang J, Liu X, Lin M, Xiao Z, Shuai X. Light-inducible nanodrug-mediated photodynamic and anti-apoptotic synergy for enhanced immunotherapy in triple-negative breast cancer. Biomater Sci. 2024;12(10): 10.1039/d4bm00083h. 10.1039/d4bm00083h 38563394

[6] Yang H, Mu W, Yuan S, Yang H, Chang L, Sang X, Gao T, Liang S, Liu X, Fu S, et al. Self-delivery photothermal-boosted-nanobike multi-overcoming immune escape by photothermal/chemical/immune synergistic therapy against HCC. J Nanobiotechnology. 2024;22(1):137. 38553725 10.1186/s12951-024-02399-3 PMC10981284

[7] Li Z, Mo F, Guo K, Ren S, Wang Y, Chen Y, Schwartz PB, Richmond N, Liu F, Ronnekleiv-Kelly SM, et al. Nanodrug-bacteria conjugates-mediated oncogenic collagen depletion enhances immune checkpoint blockade therapy against pancreatic cancer. Med. 2024;5(4):348–367.e7. 38521069 10.1016/j.medj.2024.02.012

[8] Gurung S, Khan F, Gunassekaran GR, Yoo JD, Poongkavithai Vadevoo SM, Permpoon U, Kim SH, Kim HJ, Kim IS, Han H, et al. Phage display-identified PD-L1-binding peptides reinvigorate T-cell activity and inhibit tumor progression. Biomaterials. 2020;247: Article 119984. 32278214 10.1016/j.biomaterials.2020.119984

[9] Moon Y, Shim MK, Choi J, Yang S, Kim J, Yun WS, Cho H, Park JY, Kim Y, Seong JK, et al. Anti-PD-L1 peptide-conjugated prodrug nanoparticles for targeted cancer immunotherapy combining PD-L1 blockade with immunogenic cell death. Theranostics. 2022;12(5):1999–2014. 35265195 10.7150/thno.69119 PMC8899589

[10] Qiu Z, Lu Z, Huang J, Zhong Y, Yan N, Kong R, Cheng H. Self-reinforced photodynamic immunostimulator to downregulate and block PD-L1 for metastatic breast cancer treatment. Biomaterials. 2023;303: Article 122392. 37984245 10.1016/j.biomaterials.2023.122392

[11] Chen M, Zhu Q, Zhang Z, Chen Q, Yang H. Recent advances in photosensitizer materials for light-mediated tumor therapy. Chem Asian J. 2024;19(11): Article e202400268. 38578217 10.1002/asia.202400268

[12] Yang B, Sang R, Li Y, Goldys EM, Deng W. Improved effectiveness of X-PDT against human triple-negative breast cancer cells through the use of liposomes co-loaded with protoporphyrin IX and perfluorooctyl bromide. J Mater Chem B. 2024;12(15):3764–3773. 38533806 10.1039/d4tb00011k

[13] Qiu ZW, Zhong YT, Lu ZM, Yan N, Kong RJ, Huang JQ, Li ZF, Nie JM, Li R, Cheng H. Breaking physical barrier of fibrotic breast cancer for photodynamic immunotherapy by remodeling tumor extracellular matrix and reprogramming cancer-associated fibroblasts. ACS Nano. 2024;18(13):9713–9735. 38507590 10.1021/acsnano.4c01499

[14] Xiao J, Xiao H, Cai Y, Liao J, Liu J, Yao L, Li S. Codelivery of anti-CD47 antibody and chlorin e6 using a dual pH-sensitive nanodrug for photodynamic immunotherapy of osteosarcoma. Oncol Res. 2024;32(4):691–702. 38560565 10.32604/or.2023.030767 PMC10972781

[15] Hu L, Cao Z, Ma L, Liu Z, Liao G, Wang J, Shen S, Li D, Yang X. The potentiated checkpoint blockade immunotherapy by ROS-responsive nanocarrier-mediated cascade chemo-photodynamic therapy. Biomaterials. 2019;223: Article 119469. 31520886 10.1016/j.biomaterials.2019.119469

[16] Liu H, Hu Y, Sun Y, Wan C, Zhang Z, Dai X, Lin Z, He Q, Yang Z, Huang P, et al. Co-delivery of bee venom melittin and a photosensitizer with an organic–inorganic hybrid nanocarrier for photodynamic therapy and immunotherapy. ACS Nano. 2019;13(11):12638–12652. 31625721 10.1021/acsnano.9b04181

[17] Sansaloni-Pastor S, Lange N. Unleashing the potential of 5-aminolevulinic acid: Unveiling a promising target for cancer diagnosis and treatment beyond photodynamic therapy. J Photochem Photobiol B. 2023;247: Article 112771. 37647818 10.1016/j.jphotobiol.2023.112771

[18] Wang N, Zhou Y, Yuwen X, Ren X, Zhou S, Shang Q, Jiang Y, Luan Y. Molecular engineering of anti-PD-L1 peptide and photosensitizer for immune checkpoint blockade photodynamic-immunotherapy. Chem Eng J. 2020;400:125995.

[19] Shen W, Li Y, Yang Z, Li W, Cao Y, Liu Y, Wang Z, Pei R, Xing C. Tumor microenvironment reprogramming combined with immunogenic enhancement by nanoemulsions potentiates immunotherapy. J Nanobiotechnology. 2024;22(1):154. 38581017 10.1186/s12951-024-02401-y PMC10996274

[20] Rodrigues Toledo C, Tantawy AA, Lima Fuscaldi L, Malavolta L, de Aguiar FC. EGFR- and integrin αVβ3-targeting peptides as potential radiometal-labeled radiopharmaceuticals for cancer theranostics. Int J Mol Sci. 2024;25(15):8553. 39126121 10.3390/ijms25158553 PMC11313252

[21] Pham TT, Hungnes IN, Rivas C, Cleaver J, Firth G, Blower PJ, Sosabowski J, Cook GJR, Livieratos L, Young JD, et al. Receptor-targeted peptide conjugates based on diphosphines enable preparation of ^99m^Tc and ^188^Re theranostic agents for prostate cancer. J Nucl Med. 2024;65(7):1087–1094. 38844360 10.2967/jnumed.124.267450 PMC11218721

[22] Lee CH, Bae JH, Choe EJ, Park JM, Park SS, Cho HJ, Song BJ, Baek MC. Macitentan improves antitumor immune responses by inhibiting the secretion of tumor-derived extracellular vesicle PD-L1. Theranostics. 2022;12(5):1971–1987. 35265193 10.7150/thno.68864 PMC8899590

[23] Son S, Shin JM, Shin S, Kim CH, Lee JA, Ko H, Lee ES, Jung JM, Kim J, Park JH. Repurposing macitentan with nanoparticle modulates tumor microenvironment to potentiate immune checkpoint blockade. Biomaterials. 2021;276: Article 121058. 34399119 10.1016/j.biomaterials.2021.121058

[24] Xing Y, Peng A, Yang J, Cheng Z, Yue Y, Liu F, Li F, Liu Y, Liu Q. Precisely activating cGAS-STING pathway with a novel peptide-based nanoagonist to potentiate immune checkpoint blockade cancer immunotherapy. Adv Sci. 2024;11(15):2309583. 10.1002/advs.202309583 PMC11022698 38233164

[25] Park T, Lee S, Amatya R, Cheong H, Moon C, Kwak HD, Min KA, Shin MC. ICG-loaded PEGylated BSA-silver nanoparticles for effective photothermal cancer therapy. Int J Nanomedicine. 2020;15:5459–5471. 32801700 10.2147/IJN.S255874 PMC7406329

[26] Pattipeiluhu R, Arias-Alpizar G, Basha G, Chan KYT, Bussmann J, Sharp TH, Moradi MA, Sommerdijk N, Harris EN, Cullis PR, et al. Anionic lipid nanoparticles preferentially deliver mRNA to the hepatic reticuloendothelial system. Adv Mater. 2022;34(16): Article e2201095. 35218106 10.1002/adma.202201095 PMC9461706

[27] Fang YP, Wu PC, Huang YB, Tzeng CC, Chen YL, Hung YH, Tsai MJ, Tsai YH. Modification of polyethylene glycol onto solid lipid nanoparticles encapsulating a novel chemotherapeutic agent (PK-L4) to enhance solubility for injection delivery. Int J Nanomedicine. 2012;7:4995–5005. 23055719 10.2147/IJN.S34301 PMC3457677

[28] Eguchi M, Hirata S, Ishigami I, Shuwari N, Ono R, Tachibana M, Tanuma M, Kasai A, Hashimoto H, Ogawara KI, et al. Pre-treatment of oncolytic reovirus improves tumor accumulation and intratumoral distribution of PEG-liposomes. J Control Release. 2023;354:35–44. 36586673 10.1016/j.jconrel.2022.12.050

[29] Wiedmeyer CE, Ruben D, Franklin C. Complete blood count, clinical chemistry, and serology profile by using a single tube of whole blood from mice. J Am Assoc Lab Anim Sci. 2007;46(2):59–64. 17343355

[30] Boehm O, Zur B, Koch A, Tran N, Freyenhagen R, Hartmann M, Zacharowski K. Clinical chemistry reference database for Wistar rats and C57/BL6 mice. Biol Chem. 2007;388(5):547–554. 17516851 10.1515/BC.2007.061

[31] GBD 2021 Causes of Death Collaborators. Global burden of 288 causes of death and life expectancy decomposition in 204 countries and territories and 811 subnational locations, 1990–2021: A systematic analysis for the Global Burden of Disease Study 2021. Lancet. 2024;403(10440):2100–2132. 38582094 10.1016/S0140-6736(24)00367-2 PMC11126520

[32] Mehrotra S, Sharma S, Pandey RK. A journey from omics to clinicomics in solid cancers: Success stories and challenges. Adv Protein Chem Struct Biol. 2024;139:89–139. 38448145 10.1016/bs.apcsb.2023.11.008

[33] Trivedi P, Jhala G, De George DJ, Chiu C, Selck C, Ge T, Catterall T, Elkerbout L, Boon L, Joller N, et al. TIGIT acts as an immune checkpoint upon inhibition of PD1 signaling in autoimmune diabetes. Front Immunol. 2024;15:1370907. 38533515 10.3389/fimmu.2024.1370907 PMC10964479

[34] Li Y, Li B, Wang Q, Zhang X, Zhang Q, Zhou X, Shi R, Wu Y, Zhai W, Chen Z, et al. Dual targeting of TIGIT and PD-1 with a novel small molecule for cancer immunotherapy. Biochem Pharmacol. 2024;223: Article 116162. 38527557 10.1016/j.bcp.2024.116162

[35] Jang JY, Lee BS, Huang M, Seo C, Choi JH, Shin YS, Woo HG, Kim CH. Immune checkpoint inhibitor monotherapy is sufficient to promote microenvironmental normalization via the type I interferon pathway in PD-L1-expressing head and neck cancer. Mol Oncol. 2024;18(8):1923–1939. 38511232 10.1002/1878-0261.13633 PMC11306519

[36] Zhang W, Ou M, Yang P, Ning M. The role of extracellular vesicle immune checkpoints in cancer. Clin Exp Immunol. 2024;216(3):230–239. 38518192 10.1093/cei/uxae026 PMC11097917

[37] Ghiringhelli F, Bibeau F, Greillier L, Fumet JD, Ilie A, Monville F, Laugé C, Catteau A, Boquet I, Majdi A, et al. Immunoscore immune checkpoint using spatial quantitative analysis of CD8 and PD-L1 markers is predictive of the efficacy of anti-PD1/PD-L1 immunotherapy in non-small cell lung cancer. EBioMedicine. 2023;92: Article 104633. 37244159 10.1016/j.ebiom.2023.104633 PMC10232659

[38] Peng Q, Qiu X, Zhang Z, Zhang S, Zhang Y, Liang Y, Guo J, Peng H, Chen M, Fu YX, et al. PD-L1 on dendritic cells attenuates T cell activation and regulates response to immune checkpoint blockade. Nat Commun. 2020;11(1):4835. 32973173 10.1038/s41467-020-18570-x PMC7518441

[39] Jin S, Wang H, Li Y, Yang J, Li B, Shi P, Zhang X, Zhou X, Zhou X, Niu X, et al. Discovery of a novel small molecule as CD47/SIRPα and PD-1/PD-L1 dual inhibitor for cancer immunotherapy. Cell Commun Signal. 2024;22(1):173. 38462636 10.1186/s12964-024-01555-4 PMC10926604

[40] Wang A, Xu Y, Fei Y, Wang M. The role of immunosuppressive agents in the management of severe and refractory immune-related adverse events. Asia Pac J Clin Oncol. 2020;16(4):201–210. 32212243 10.1111/ajco.13332

[41] Li Y, Liu J, Gao L, Liu Y, Meng F, Li X, Qin FX. Targeting the tumor microenvironment to overcome immune checkpoint blockade therapy resistance. Immunol Lett. 2020;220:88–96. 30885690 10.1016/j.imlet.2019.03.006

[42] Dai X, Du Y, Li Y, Yan F. Nanomaterials-based precision sonodynamic therapy enhancing immune checkpoint blockade: A promising strategy targeting solid tumor. Mater Today Bio. 2023;23: Article 100796. 10.1016/j.mtbio.2023.100796 PMC10520454 37766898

[43] Sun Y, Lyu B, Yang C, He B, Zhang H, Wang X, Zhang Q, Dai W. An enzyme-responsive and transformable PD-L1 blocking peptide-photosensitizer conjugate enables efficient photothermal immunotherapy for breast cancer. Bioact Mater. 2022;22:47–59. 36203955 10.1016/j.bioactmat.2022.08.020 PMC9519467

[44] Chang R, Li T, Fu Y, Chen Z, He Y, Sun X, Deng Y, Zhong Y, Xie Z, Yang Y, et al. A PD-L1 targeting nanotheranostic for effective photoacoustic imaging guided photothermal-immunotherapy of tumor. J Mater Chem B. 2023;11(35):8492–8505. 37594411 10.1039/d3tb00221g

[45] Li L, Fu J, Ye J, Liu L, Sun Z, Wang H, Tan S, Zhen M, Wang C, Bai C. Developing hypoxia-sensitive system via designing tumor-targeted fullerene-based photosensitizer for multimodal therapy of deep tumor. Adv Mater. 2024;36(23): Article e2310875. 38450765 10.1002/adma.202310875

[46] Chen Y, Shu X, Guo JY, Xiang Y, Liang SY, Lai JM, Zhou JY, Liu LH, Wang P. Nanodrugs mediate TAMs-related arginine metabolism interference to boost photodynamic immunotherapy. J Control Release. 2024;367:248–264. 38272398 10.1016/j.jconrel.2024.01.045

[47] Lee JH, Yang SB, Park SJ, Kweon S, Ma G, Seo M, Kim HR, Kang TB, Lim JH, Park J. Cell-penetrating peptide like anti-programmed cell death-ligand 1 peptide conjugate-based self-assembled nanoparticles for immunogenic photodynamic therapy. ACS Nano. 2025;19(2):2870–2889. 39761412 10.1021/acsnano.4c16128

[48] Zheng J, Huang J, Zhang L, Wang M, Xu L, Dou X, Leng X, Fang M, Sun Y, Wang Z. Drug-loaded microbubble delivery system to enhance PD-L1 blockade immunotherapy with remodeling immune microenvironment. Biomater Res. 2023;27(1):9. 36759928 10.1186/s40824-023-00350-5 PMC9909878

[49] Mei KC, Thota N, Wei PS, Yi B, Bonacquisti EE, Nguyen J. Calreticulin P-domain-derived “eat-me” peptides for enhancing liposomal uptake in dendritic cells. Int J Pharm. 2024;653: Article 123844. 38272193 10.1016/j.ijpharm.2024.123844 PMC10994729

