# Supplementary material for: Dual-Active Nanoimmunomodulators for the Synergistic Enhancement of the Antitumor Efficacy of Photodynamic Immunotherapy
Source: Biomater Res. 2025 Jun 9;29:0214. doi: 10.34133/bmr.0214 (PMC12147445; doi:10.34133/bmr.0214)
Supplement: Supplementary 1 — Figs. S1 to S16 [file bmr.0214.f1.docx]

# Supplemental Material


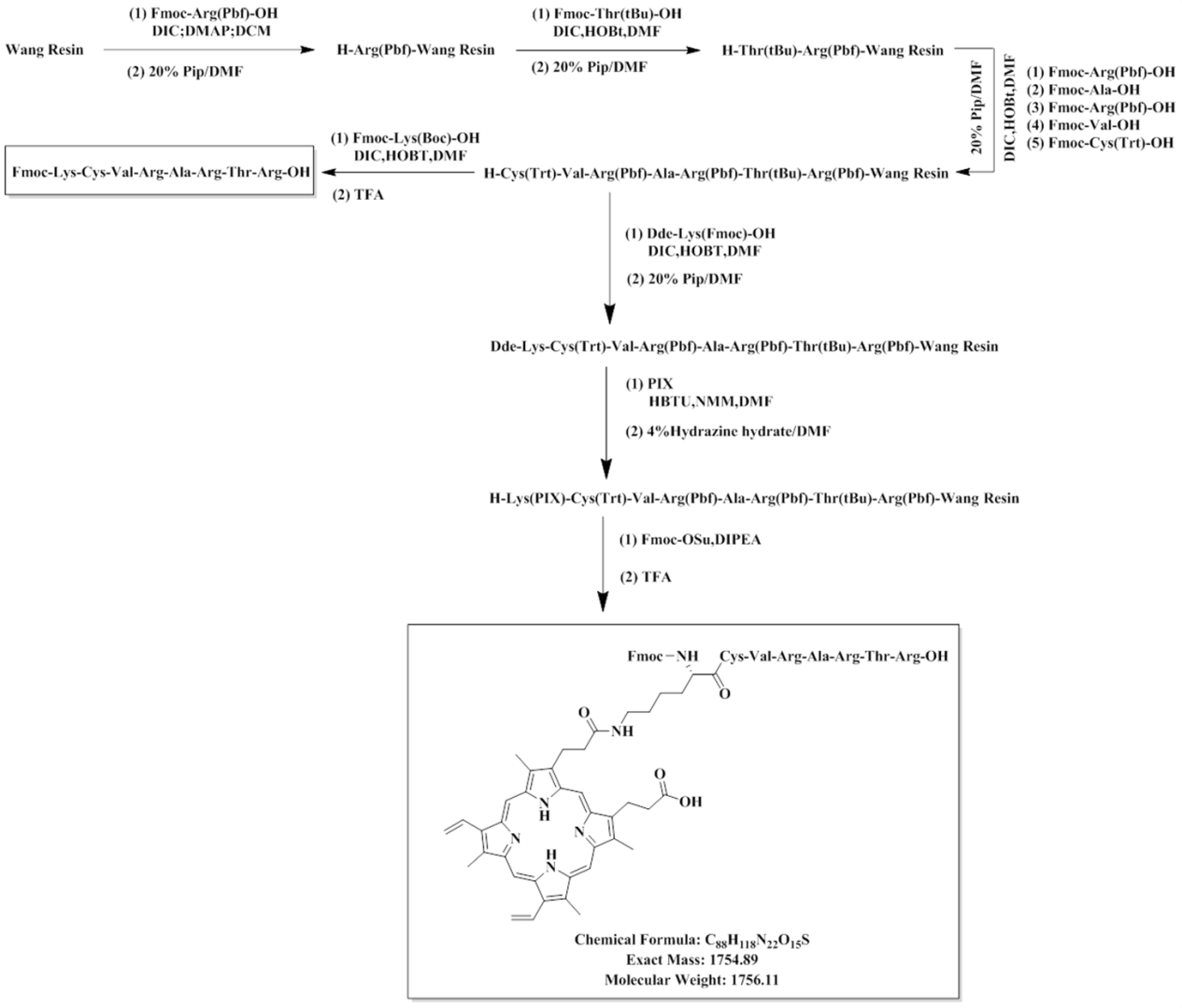


**Fig. S1**. Synthesis roadmap for CP. First, Dde - Lys - Cys (Trt) - Val - Arg (Pbf) - Ala - Arg (Pbf) - Thr (tBu) - Arg (pbf) - Wang Resin was obtained by solid-phase synthesis of peptides; then, PIX was added to obtain H - Lys (PIX) - Cys (Trt) - Val - Arg (Pbf) - Ala - Arg (Pbf) - Thr (tBu) - Arg (pbf) - Wang Resin; finally, after the reaction of feeding Fmoc-Osu and DIPEA, TFA cutting solution was added, and the target product CP was obtained after purification


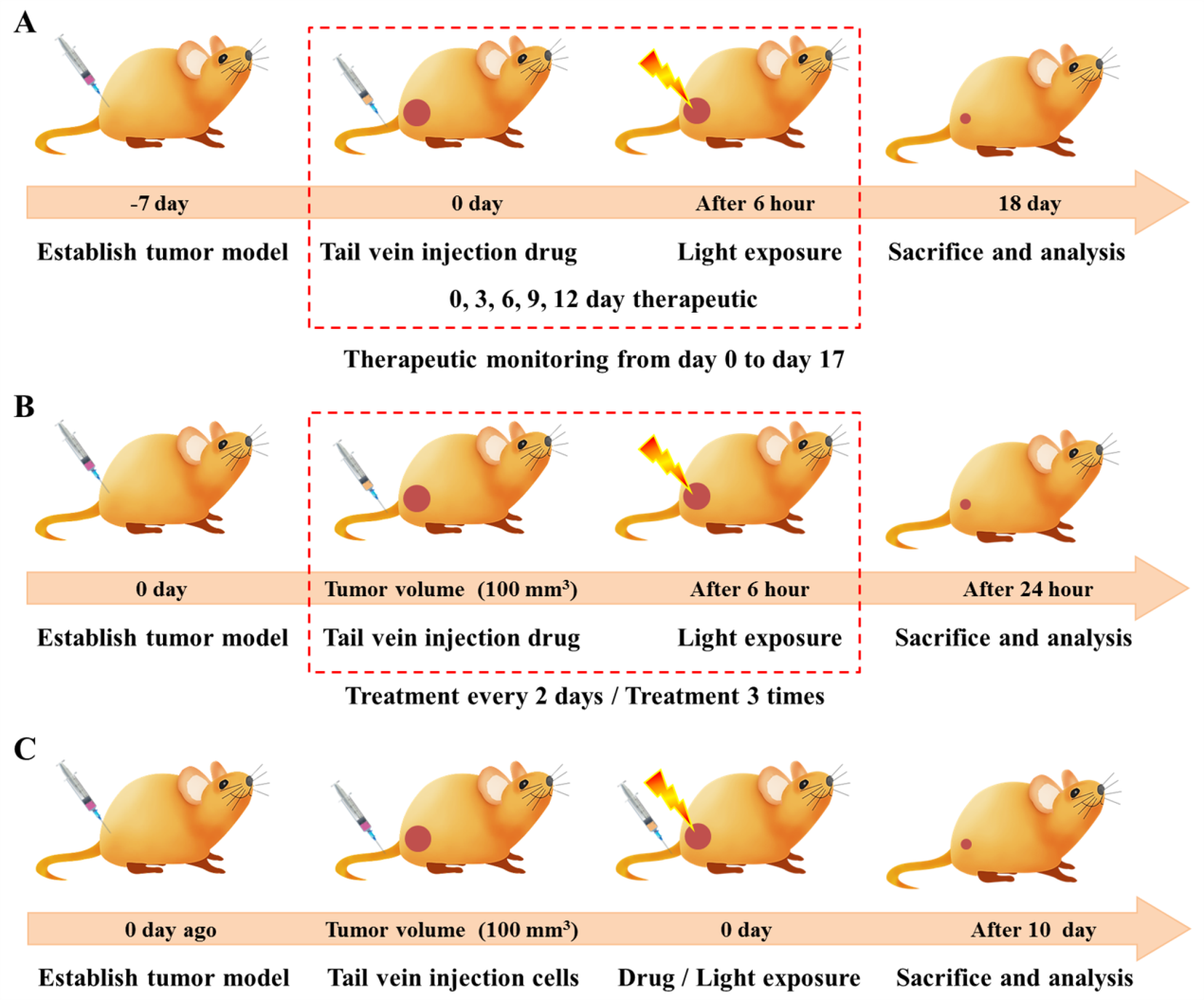


**Fig. S2**. Flowchart of the construction and treatment of mouse tumor models. A. Model construction and treatment flowchart for tumor growth inhibition study; B. Animal model construction and treatment flowchart for immune response study; C. Animal model construction and treatment flowchart for lung metastasis study


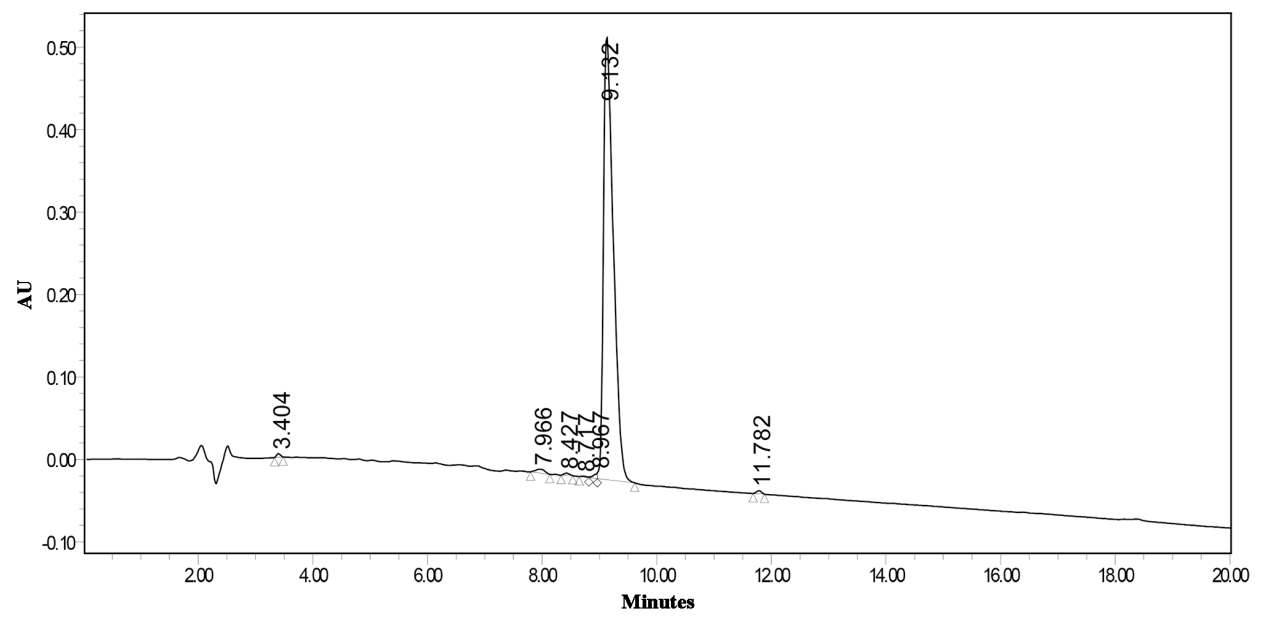


**Fig. S3**. HPLC results of CP


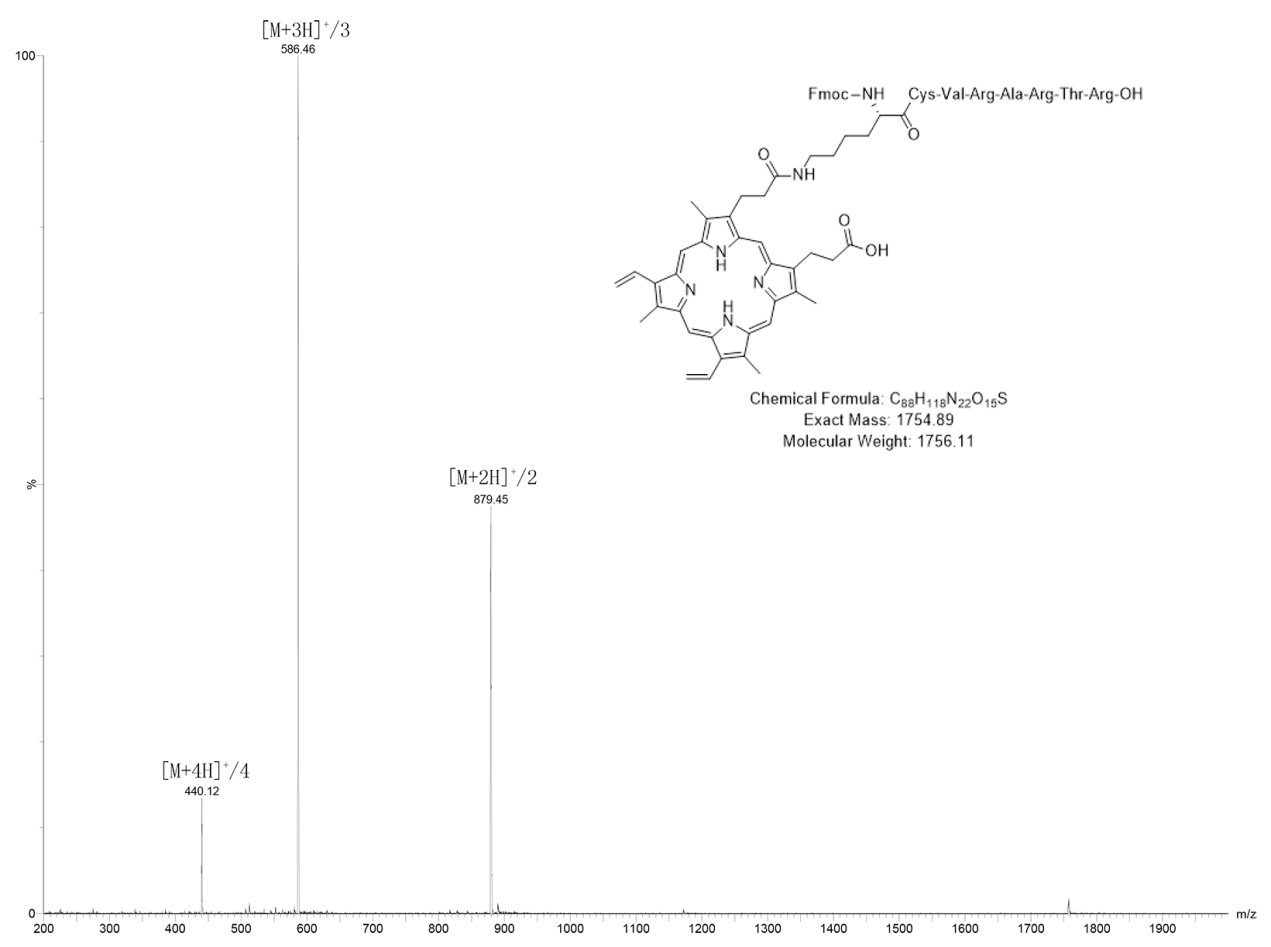


**Fig. S4**. Results of mass spectrometry analysis of CP


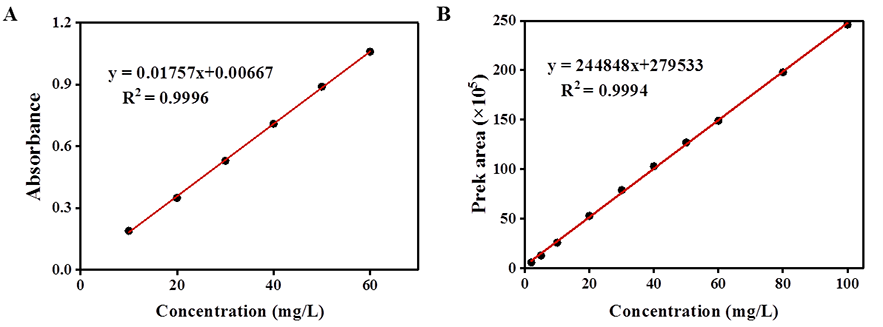


**Fig. S5**. Standard curves for PIX and MAC. A. UV-Vis test results plotting the standard curve for PIX; B. HPLC test results plotting the standard curve for MAC


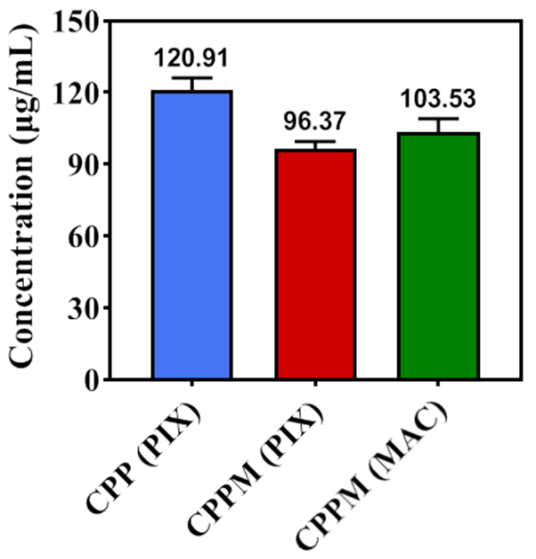


**Fig. S6**. Concentration tests for PIX and MAC in CPP and CPPM


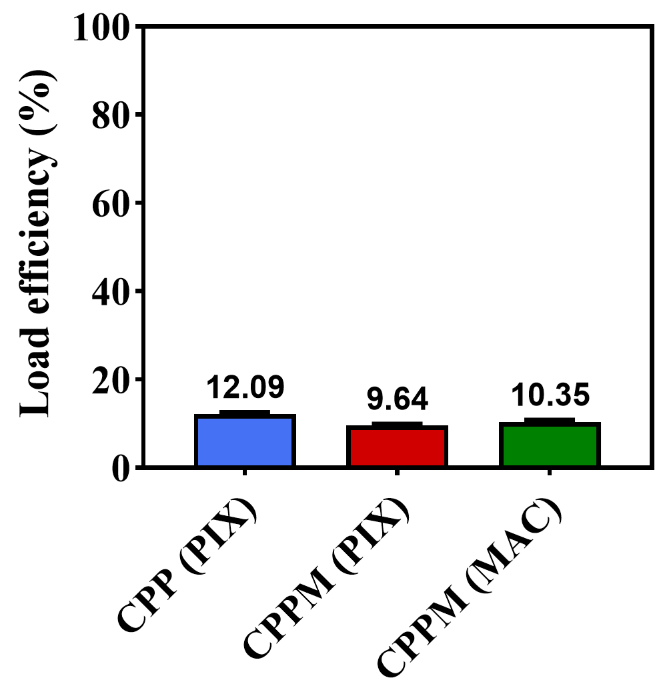


Fig. S7. Load efficiency tests for PIX and MAC in CPP and CPPM


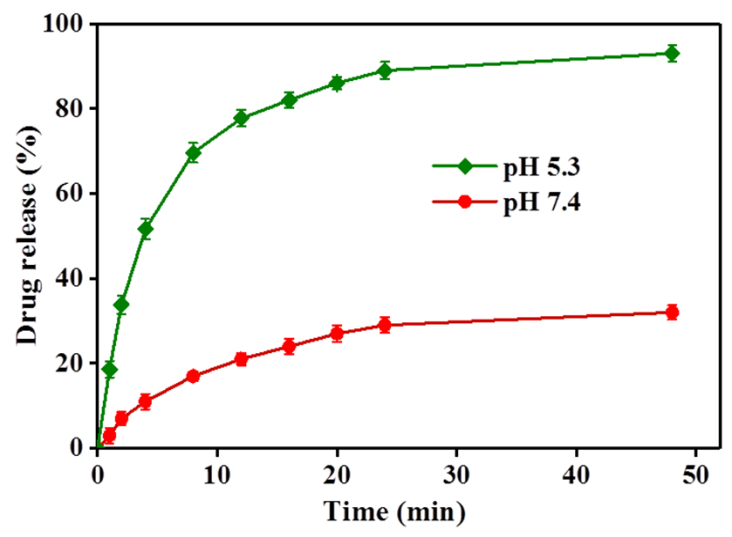


**Fig. S8**. Release profiles of MAC from CPPM at different pH conditions


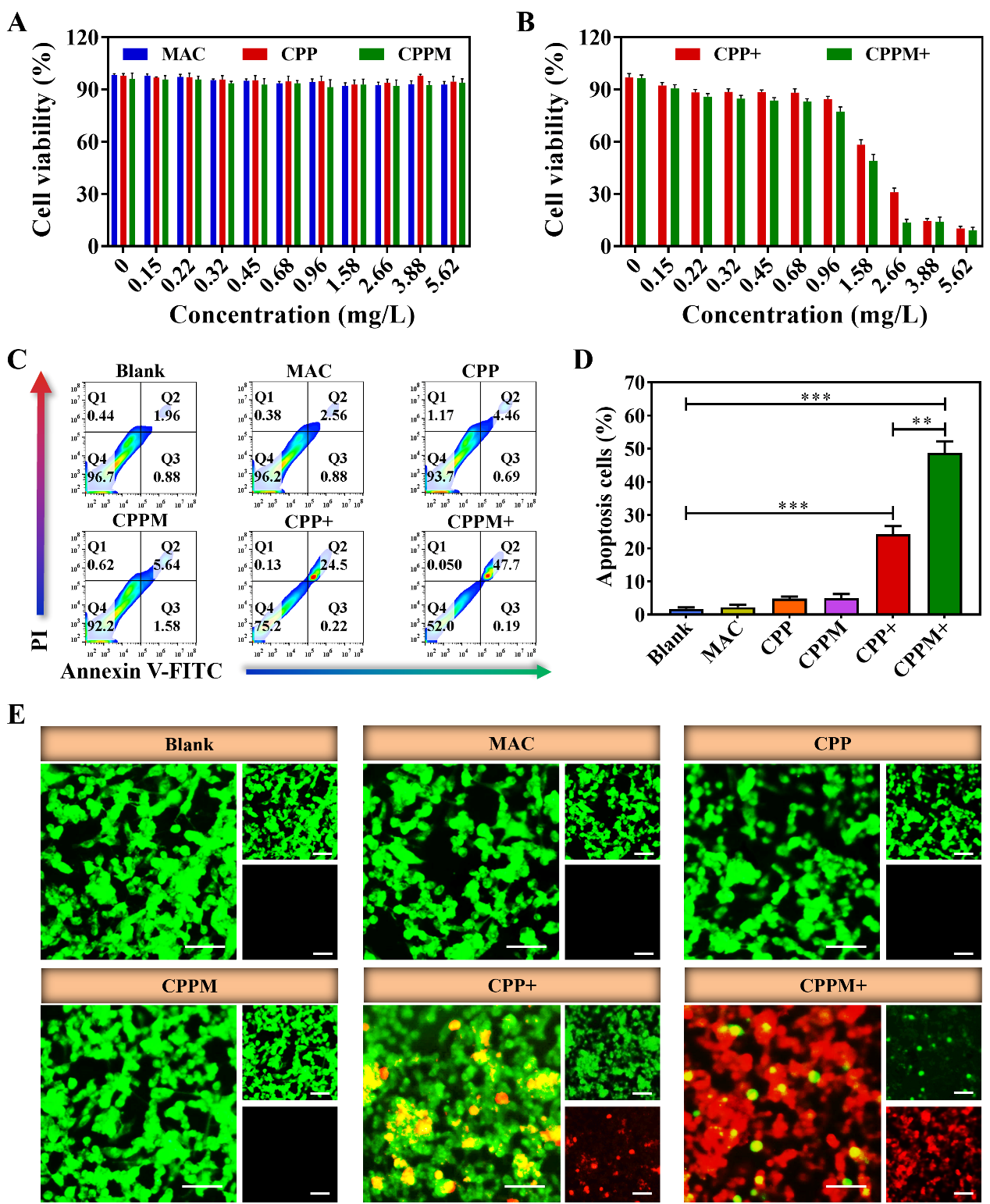


**Fig. S9**. In vitro anti-tumor effects of CPPM. A. MTT method to detect the survival rate of CT26 cells under dark treatment conditions for MAC, CPP, and CPPM; B. MTT method to detect the survival rate of CT26 cells under light treatment for CPP, CPPM; C. Quantification of the percentage of apoptosis of CT26 cells by MAC, CPP, CPPM in light and no light conditions in flow cytometry; D. Apoptosis of CT26 cells after uptake of MAC, CPP, CPPM in light versus no light condition; E. Staining results of CT26 cells after uptake of MAC, CPP, and CPPM by microscopy of live and dead cells. Scale bar: 20 μm


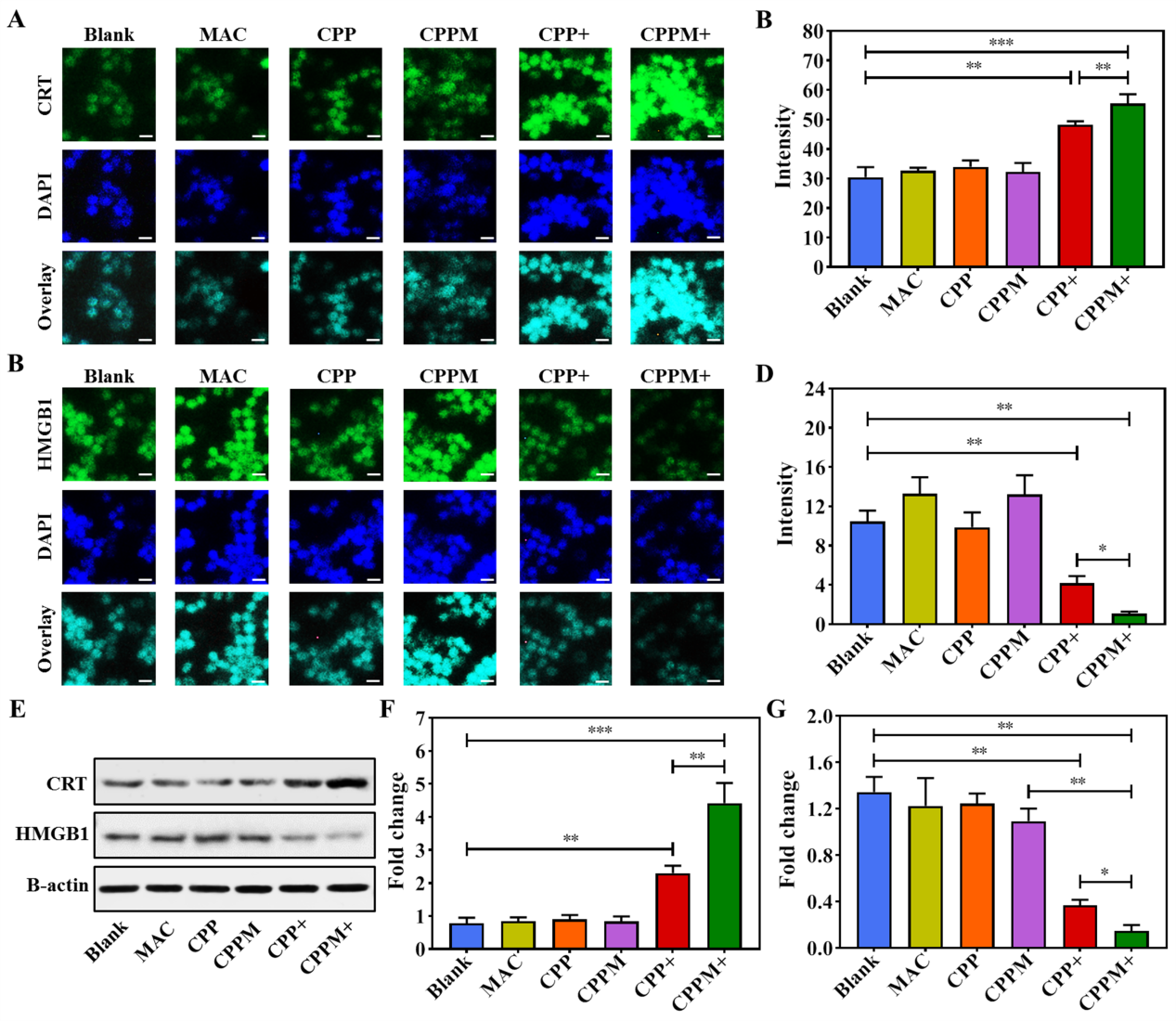


**Fig. S10**. Study of the mechanism of action of PDT-induced ICD. A. Immunofluorescence staining of CRT detected after co-culture of CT26 cells with MAC, CPP, and CPPM in light or no light treatment; B. Quantitative fluorescence analysis of CRT; C. Immunofluorescence staining of HMGB1 detected after co-culture of CT26 cells with MAC, CPP, and CPPM in light or no light treatment; D. Quantitative fluorescence analysis of HMGB1; E. Detection of HMGB1 and CRT proteins after co-culture of CT26 cells with MAC, CPP, and CPPM with or without light treatment; F. Quantitative protein analysis of HMGB1; G. Quantitative protein analysis of CRT. Scale bar: 10 μm.


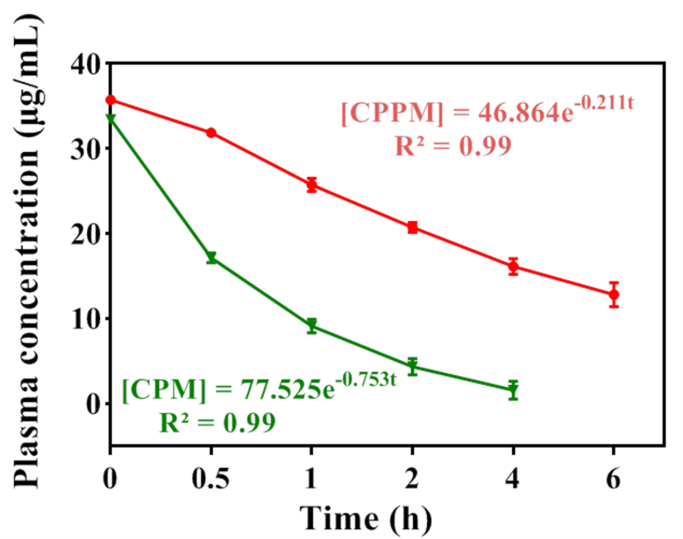


**Fig. S11**. Pharmacokinetic profile of CPPM


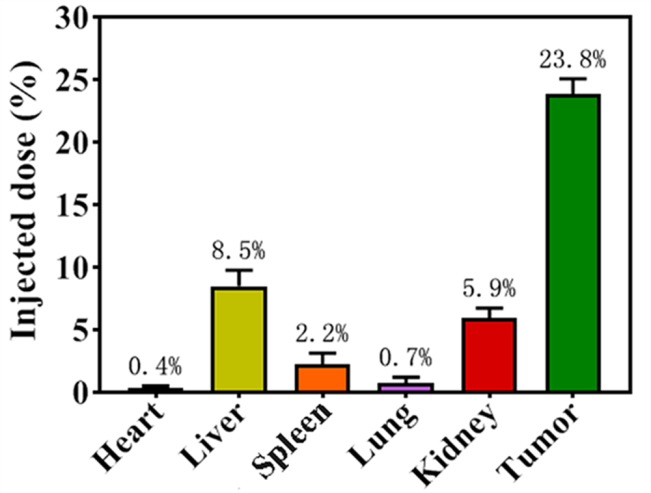


**Fig. S12**. Cumulative content of CPPM in each organ and tumor tissue 6 hours after drug injection as a percentage of the injected dose


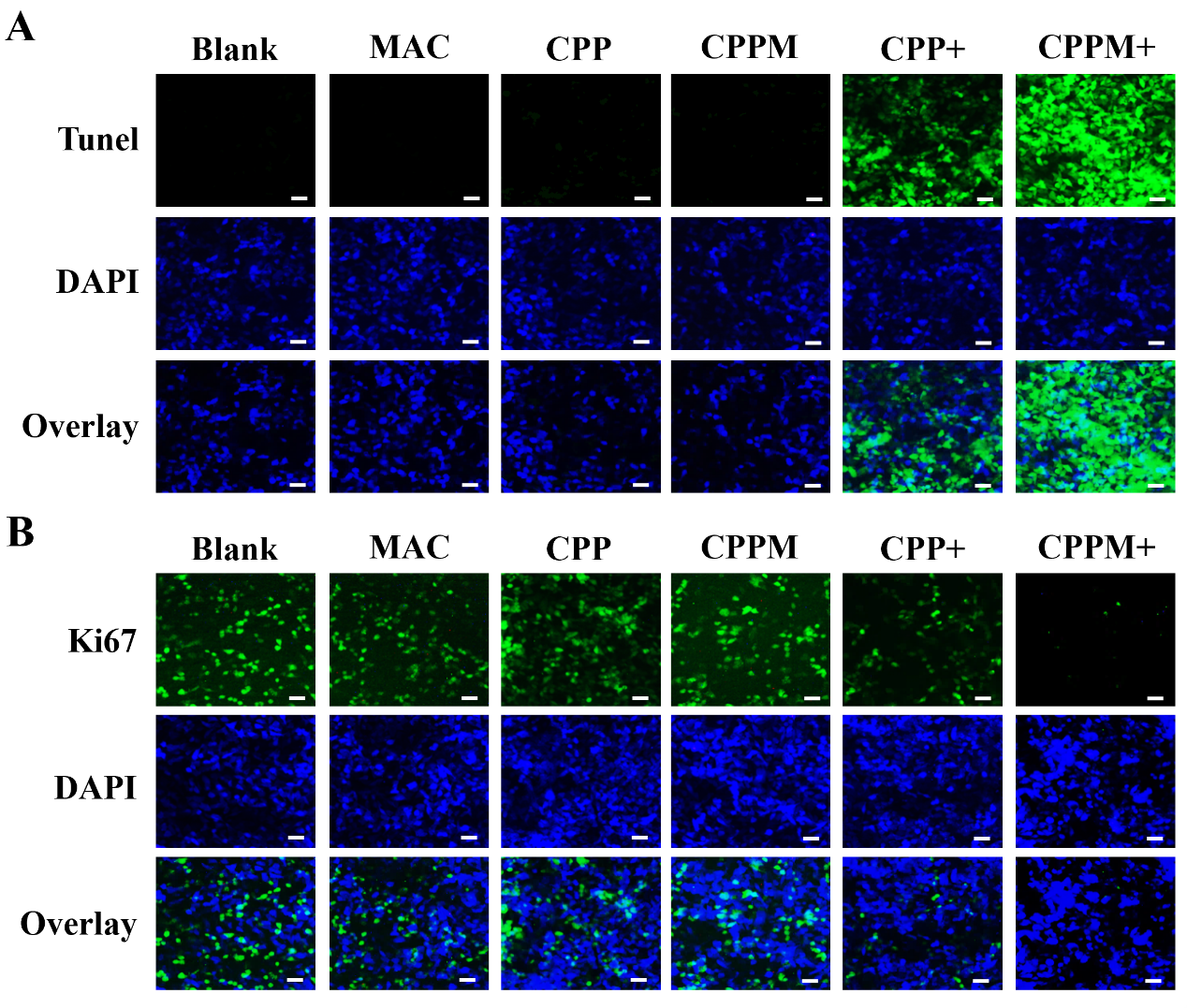


**Fig. S13**. I. Analysis of Tunel staining of tumors in each group after day 17; J. Ki67 staining analysis of tumors in each group after day 17. Scale bar: 50 μm
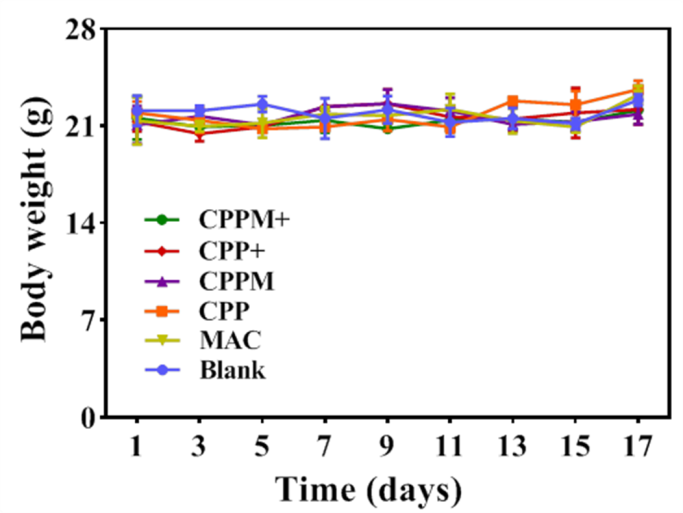


**Fig. S14**. Comparison of body weights of mice in each group


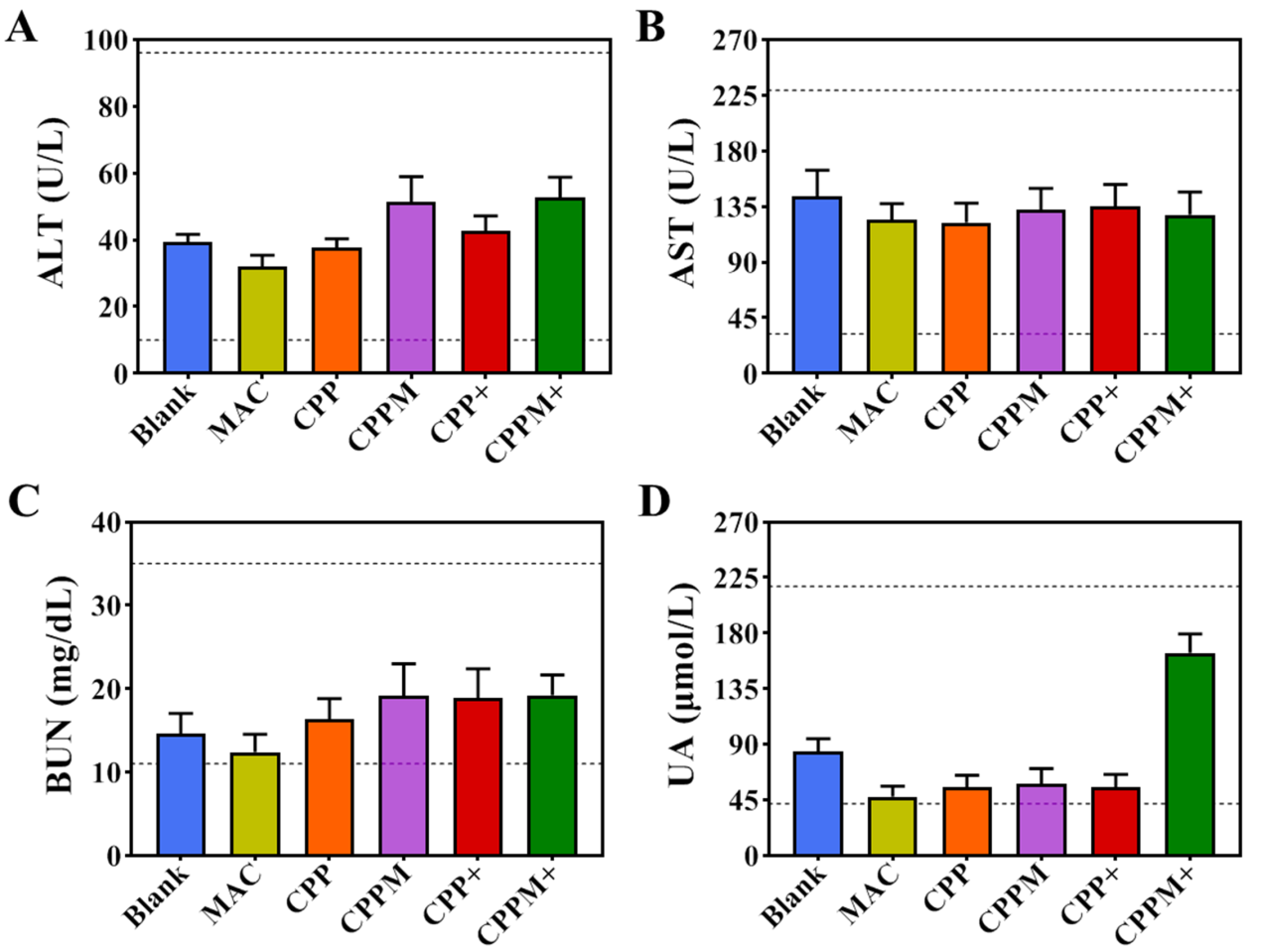


**Fig. S15**. Mouse liver function and kidney function related indexes assay. A. Liver function indicator ALT assay; B. Liver function indicator AST assay; C. Kidney function indicator BUN detection; D. Renal function indicator UA assay. The dotted line indicates the normal reference value range


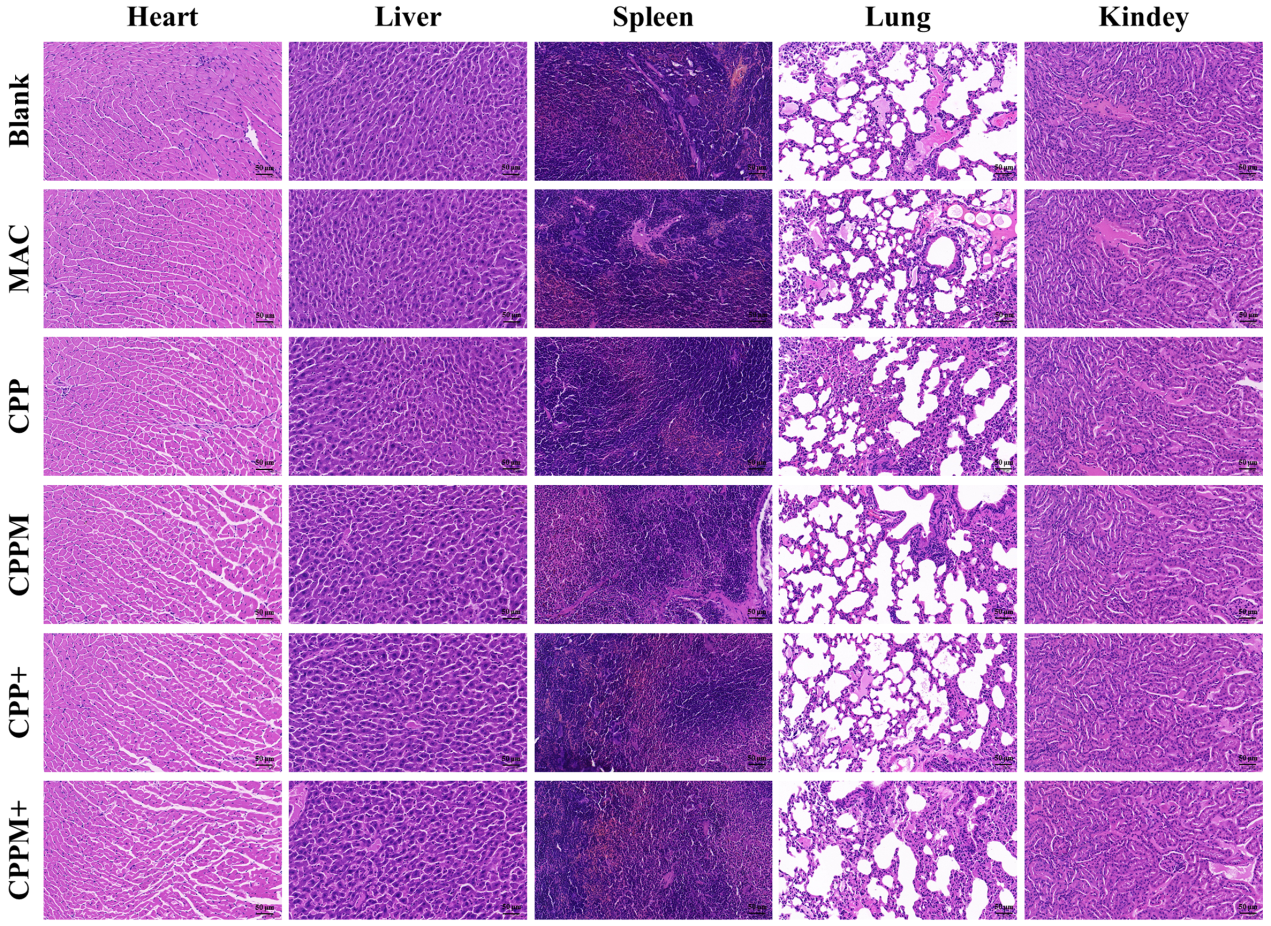


**Fig. S16**. H&E staining of heart, liver, spleen, lungs, and kidneys of various groups of mice
